# Galectin-9 has non-apoptotic cytotoxic activity toward acute myeloid leukemia independent of cytarabine resistance

**DOI:** 10.1038/s41420-023-01515-w

**Published:** 2023-07-06

**Authors:** Ghizlane Choukrani, Nienke Visser, Natasha Ustyanovska Avtenyuk, Mirjam Olthuis, Glenn Marsman, Emanuele Ammatuna, Harm Jan Lourens, Toshiro Niki, Gerwin Huls, Edwin Bremer, Valerie R. Wiersma

**Affiliations:** 1 Department of Hematology, University Medical Center Groningen, University of Groningen, Groningen, The Netherlands; 2 Surflay Nanotec GmbH, Berlin, Germany; 3 Department of Immunology, Kagawa University, Takamatsu, Kagawa Japan

**Keywords:** Acute myeloid leukaemia, Cancer therapeutic resistance, Macroautophagy, Glycobiology

## Abstract

Acute myeloid leukemia (AML) is a malignancy still associated with poor survival rates, among others, due to frequent occurrence of therapy-resistant relapse after standard-of-care treatment with cytarabine (AraC). AraC triggers apoptotic cell death, a type of cell death to which AML cells often become resistant. Therefore, therapeutic options that trigger an alternate type of cell death are of particular interest. We previously identified that the glycan-binding protein Galectin-9 (Gal-9) has tumor-selective and non-apoptotic cytotoxicity towards various types of cancer, which depended on autophagy inhibition. Thus, Gal-9 could be of therapeutic interest for (AraC-resistant) AML. In the current study, treatment with Gal-9 was cytotoxic for AML cells, including for CD34^+^ patient-derived AML stem cells, but not for healthy cord blood-derived CD34^+^ stem cells. This Gal-9-mediated cytotoxicity did not rely on apoptosis but was negatively associated with autophagic flux. Importantly, both AraC-sensitive and -resistant AML cell lines, as well as AML patient samples, were sensitive to single-agent treatment with Gal-9. Additionally, Gal-9 potentiated the cytotoxic effect of DNA demethylase inhibitor Azacytidine (Aza), a drug that is clinically used for patients that are not eligible for intensive AraC treatment. Thus, Gal-9 is a potential therapeutic agent for the treatment of AML, including AraC-resistant AML, by inducing caspase-independent cell death.

## Introduction

Acute myeloid leukemia (AML) is an aggressive hematologic malignancy characterized by the accumulation of various cytogenetic and molecular alterations [1, 2]. The standard treatment regimen of AML patients comprises the nucleoside analog cytarabine (AraC) combined with an anthracycline such as daunorubicin or idarubicin. However, prolonged disease-free survival following therapy with AraC is uncommon due to the development of therapy resistance [3]. Consequently, the 5-year overall survival rate for AML is just 25%, although there is a significant variability depending on the AML subtype, ranging from 5 to 10% overall survival for individuals with poor-risk AML to over 90% overall survival for patients with acute promyelocytic leukemia [4].

One of the primary mechanisms underlying AraC resistance in AML is an evasion of the apoptotic cell death program, with, e.g., deregulated expression of proteins that regulate apoptosis [5–7]. Thus, novel therapeutic strategies that do not rely on apoptosis are of particular interest for the treatment of relapsed patients with AraC-resistant AML. Furthermore, patients that are not eligible for intensive chemotherapy, particularly elderly patients (>65 years of age) and those with intermediate-risk cytogenetics, are treated with the hypomethylating agent azacitidine (Aza) [8]. However, the survival rate of those patients after Aza treatment is merely 14 months [8], making the search for more effective treatment options for these patients warranted.

An interesting candidate in this respect is Galectin-9 (Gal-9), a carbohydrate-binding protein that is comprised of two CRDs connected by an inter-domain linker [9]. We and others have previously demonstrated that a recombinant form of Gal-9 has potent cytotoxic activity toward various cancer types [10–14]. Furthermore, Gal-9 killed imatinib-resistant chronic myelogenous leukemia cells [12]. Although associated with features of apoptosis, like phosphatidylserine exposure, Gal-9-induced cytotoxicity could not be blocked by pan-caspase inhibition and thus did not require apoptotic signaling [13, 14]. Instead, cytotoxicity is associated with the induction and halted execution of its proper execution [14]. Of note, whereas Gal-9 had potent cytotoxic activity toward cancer cells, it did not negatively affect the viability of healthy counterparts [13, 14]. Based on this non-apoptotic cytotoxic activity of Gal-9, we hypothesized that Gal-9 might also be of potential interest for the treatment of AML in general, as well as for therapy-resistant AML.

In the current study, treatment with recombinant Gal-9 induced cell death in both AML cell lines and primary patient-derived AML cells, including CD34^+^ AML stem cells, but not healthy CD34^+^ cord blood (CB)-derived stem cells. Furthermore, AraC-sensitive as well as AraC-resistant AMLs were equally susceptible to Gal-9 cytotoxicity. Gal-9-mediated cytotoxicity towards AML cells did not rely on apoptotic signaling but was associated with halted execution of autophagy. Finally, the combination of Gal-9 with Aza induced more cell death compared to either Aza or Gal-9 alone. Thus, Gal-9 may be a potential novel therapeutic agent for the treatment of AML, including AraC-resistant AML.

## Results

### Galectin-9 is cytotoxic for AML cell lines

**Fig. 1:**
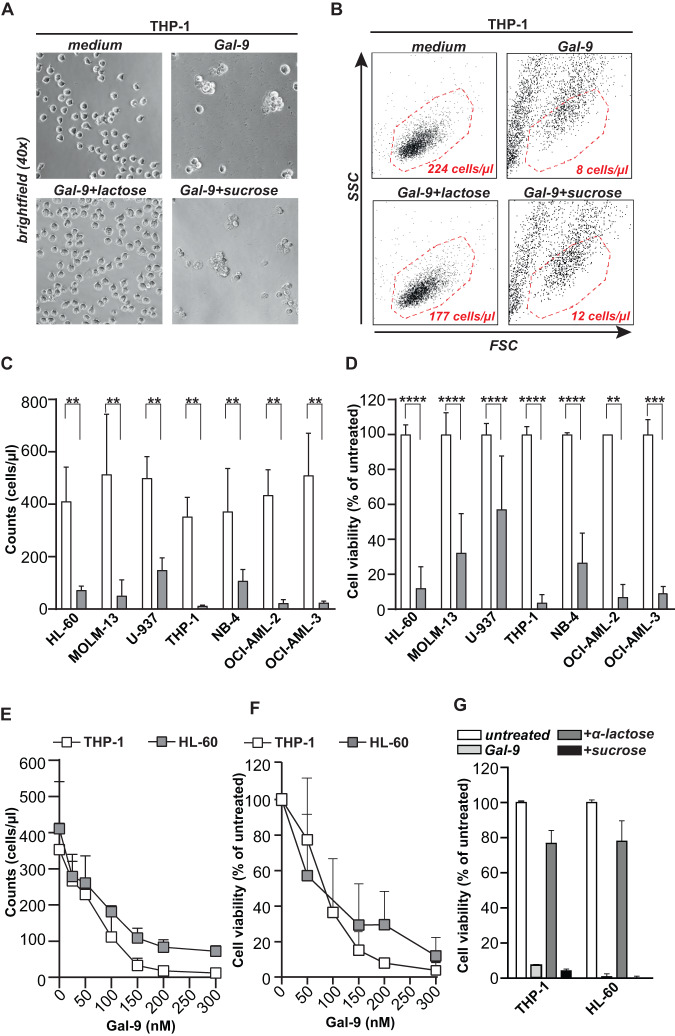
Gal-9 is cytotoxic for AML cell lines. Viability of the THP-1 cell line treated with 300 nM Gal-9 for 16 h, visualized by microscopy (**A**) or (**B**) flow cytometry. **C** Flow cytometry-based cell counts or **D** cell viability using the MTS assay upon treatment of different AML cell lines with Gal-9 (300 nM) for 16 or 72 h, respectively. **E** Dose-dependent impact of Gal-9 on flow cytometry-based cell counts or **F** cell viability upon treatment with the indicated concentrations of Gal-9 for 72 h (*n* = 5). **G** Percentage of cell viability upon treatment with 300nM Gal-9 in the presence of α-lactose or sucrose (40 mM) (*n* = 10).

Previously, we identified that treatment of various solid cancers with recombinant Gal-9 induced cancer-specific cell death [13, 14]. In line with this finding, ‘short-term’ treatment of a panel of AML lines with Gal-9 (300 nM) strongly and significantly reduced the number of viable cells, as visualized using microscopy for THP-1 (Fig. 1A). Upon quantification using flow cytometry, Gal-9 treatment strongly reduced viable counts of the THP-1 cell line (Fig. 1B), and additional AML cell lines (Fig. 1C). This effect was dose-dependent (Fig. 1E, Supplementary Fig. 1A–E) with EC50 for cell lines ranging from 92 to 193 nM for cell counts (Supplementary Fig. 1K). Correspondingly, treatment with Gal-9 significantly and dose-dependently reduced the viability of AML cells (Fig. 1D, F and Supplementary Fig. 1F–J), with EC50s ranging from 115 to 300 nM (Supplementary Fig. 1K). This cytotoxic activity of Gal-9 was inhibited by co-incubation with the carbohydrate recognition domain (CRD)-blocking sugar α-lactose, but not by the irrelevant sugar sucrose, demonstrating CRD-dependent activity (Fig. 1G and Supplementary Fig. 1L, M). Thus, Gal-9 had direct and dose-dependent cytotoxic activity toward AML cells.

### Galectin-9 is cytotoxic for patient-derived CD34^+^ AML stem cells and CD34^−^ AML blasts but not for CD34^+^ CB-derived stem cells

**Fig. 2:**
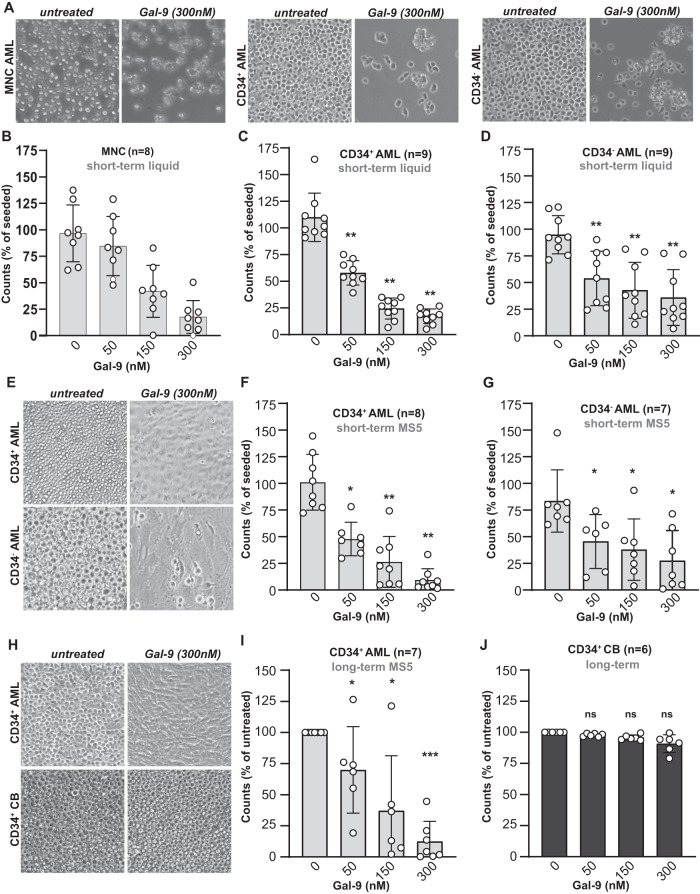
Gal-9 is cytotoxic for patient-derived AML cells but not CD34^+^ cord blood-derived cells. **A** Images of Gal-9-treated patient-derived AML, including MNC AML, CD34^+^ AML, and CD34^−^ AML cells (light microscopy). Dose-dependent cytotoxicity of Gal-9 in liquid culture (1–3 days) for patient-derived AML cells in either the **B** MNC AML, **C** CD34^+^ AML, or **D** CD34^−^ AML cell fraction. **E** Pictures of CD34^+^ patient-derived AML cells treated with Gal-9 (150, 300 nM) and CQ (20, 50 µM) for 4 days on top of an MS5 monolayer (light microscopy). Short-term (16 h) treatment of **F** patient-derived CD34^+^ or **G** CD34^−^ AML cells on an MS5 stromal layer using the indicated concentrations of Gal-9. **H** Pictures of CD34^+^ patient-derived AML cells treated with Gal-9 (150, 300 nM) and CQ (20, 50 µM) for 4 days on top of an MS5 monolayer (light microscopy). Long-term incubation (5–7 days) of **I** CD34^+^ AML or **J** CD34^+^ CB cells on top of an MS5 stromal layer with the indicated doses of Gal-9 and evaluated using flow cytometry-based counts.

The cytotoxicity of Gal-9 was further assessed toward patient-derived AML cells (see Supplementary Table 1 for patient characteristics), with a clear reduction in cell density in both unsorted, CD34^+^ AML stem cell and CD34^−^ AML blast populations after treatment with Gal-9 (Fig. 2A). This effect was again CRD-specific as α-lactose abrogated cytotoxicity (Supplementary Fig. 2A) and was dose-dependent (Fig. 2B–D). Notably, cytotoxicity towards CD34^+^ AML cells was slightly higher than toward CD34^−^ AML, albeit not significantly (Supplementary Fig. 2B). Nevertheless, treatment with a low dose of Gal-9 reduced the percentage of CD34^+^ cells in a mixed culture with CD34^-^ cells (Supplementary Fig. 2C, D).

In the bone marrow niche, AML cells are surrounded and supported by stromal cells, with in vitro co-cultures typically being less sensitive to treatment [15]. However, short-term treatment of AML/MS5 stromal cell co-cultures with Gal-9 almost completely eliminated CD34^+^ as well as CD34^−^ AML cells from co-cultures, with only a monolayer of unaffected MS5 remaining (Fig. 2E, see also data in Fig. 5). In line with this, Gal-9 treatment of MS5 cells alone did not impact on viability (Supplementary Fig. 2E). In a panel of patient-derived AML cells in co-culture with MS5, Gal-9 dose-dependently reduced viability by ~90% for CD34^+^ AML and ~75% for CD34^−^ AML, respectively (Fig. 2F, G). No significant difference in sensitivity was detected between the AML stem cell and blast population (Supplementary Fig. 2F), although the cytotoxic effect of Gal-9 in AML/MS5 cocultures was significantly higher than in liquid cultures without stromal support (Supplementary Fig. 2G). Again, Gal-9 cytotoxicity was abrogated by co-incubation with α-lactose (Supplementary Fig. 2H).

Importantly, treatment with a single dose of Gal-9 eliminated the CD34^+^ AML cells even after ‘long-term’ incubation of up to 7 days, whereas CB-derived CD34^+^ stem cells remained unaffected, as shown by microscopy images (Fig. 2H). The cytotoxic effect of Gal-9 on CD34^+^ AML cells in these longer-term co-cultures was dose-dependent (Fig. 2I), not affecting CD34^+^ CB cells at any concentration (Fig. 2J), yielding the strongest differential effects at 300 nM Gal-9 (Supplementary Fig. 2I). In addition, whereas Gal-9 reduced mitochondrial membrane potential as measured by DioC6 in CD34^+^ AML cells, this effect was not observed in CD34^+^ CB cells (Supplementary Fig. 2J). Like for CD34^+^ AML cells, Gal-9 dose-dependently eliminated CD34^−^ AML cells in long-term assays (Supplementary Fig. 2K). Notably, repeated treatment with a low dose of Gal-9 (25–50 nM) every 3rd day yielded a similar reduction in cell counts as a single dose with 300 nM Gal-9 after 2 weeks (Supplementary Fig. 2L). Taken together, Gal-9 had a dose-dependent cytotoxic effect towards patient-derived CD34^+^ as well as CD34^-^ cells in both liquid and MS5 co-cultures, whereas it did not negatively impact on MS5 stromal cells and healthy CB-derived CD34^+^ stem cells.

### Galectin-9-induced AML cell death did not rely on caspase-dependent apoptosis

**Fig. 3:**
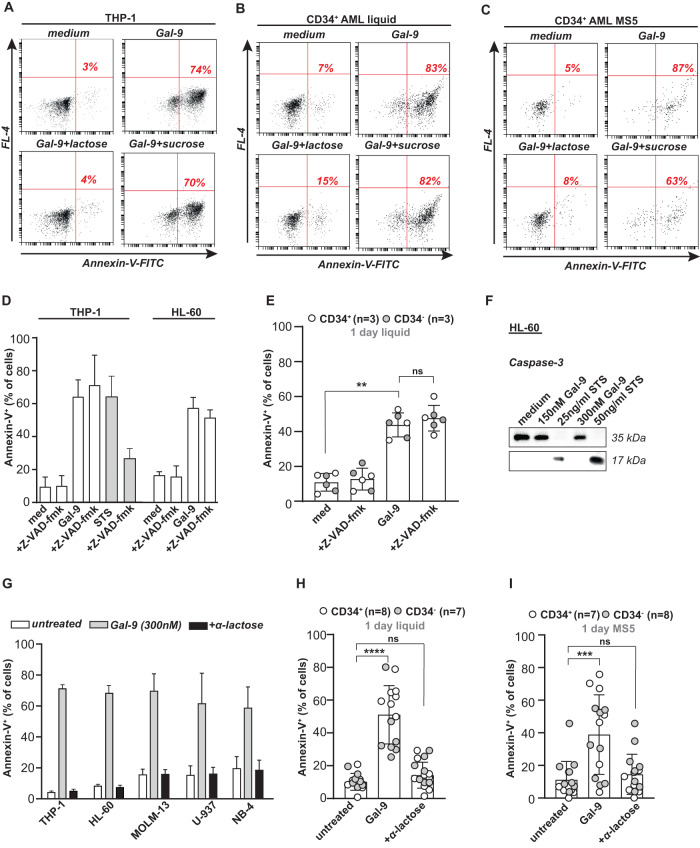
Gal-9-induced AML cell death does not rely on caspase-dependent apoptosis. PS-exposure detected by flow cytometry in Gal-9-treated (300 nM, 16 h). **A** THP-1 cells, CD34^+^ patient-derived AML cells in **B** liquid, or **C** MS5 co-cultures. **D** Quantification of PS exposure after blockade of pan-caspases with Z-VAD-FMK in Gal-9-treated THP-1 and HL-60 cells, using staurosporine (STS) as a positive control (*n* = 3). **E** As in **D** but determined on patient-derived CD34^+^ and CD34^-^ cells. **F** Western blot of full-length caspase-3 (35 kDa) and cleaved caspase-3 (17 kDa) in HL-60 cells treated with Gal-9 (150, 300 nM, 16 h) or STS (25, 50 ng/ml, 16 h). Blockade of Gal-9-mediated PS-exposure with α-lactose (40 mM) in (**G**) a panel of AML cell lines (*n* = 5), **H** CD34^+^/CD34^−^ patient-derived AML cells in liquid culture, or **I** in co-culture with MS5.

In several reports, Gal-9-induced cell death was reported to rely on apoptotic signaling [16–18]. In contrast, we previously demonstrated that Gal-9-induced cell death, although being characterized by the apoptotic feature phosphatidylserine (PS)-exposure, was caspase-independent in colon cancer and melanoma cells [13, 14]. Also, in AML, PS exposure was detected upon Gal-9 treatment of THP-1 cells and CD34^+^ patient-derived AML cells in both liquid and MS5 co-cultures (Fig. 3A–C). However, this effect was not blocked by co-incubation with the pan-caspase inhibitor Z-VAD-FMK in cell lines (Fig. 3D) nor patient-derived AML cells (Fig. 3E). In contrast, Z-VAD-FMK did reduce PS-exposure upon treatment with apoptosis-inducer staurosporine (Fig. 3D). Furthermore, no processing of caspase-3 was detected in Gal-9-treated HL-60 cells, whereas staurosporine did induce caspase-3 processing (Fig. 3F). Like the reduction in cell counts and cell viability, PS-exposure was dose-dependent (Supplementary Fig. 3A–G), with an EC50 ranging from 77 to 140 nM in the cell line panel (Suppl. Fig. 3H), and CRD-dependent (Fig. 3G–I). PS-exposure was less pronounced in MS5 co-cultures and especially in CD34^−^ AML cells, only reaching significance at 300 nM Gal-9 (Supplementary Fig. 3I–L). Furthermore, Gal-9 treatment did not induce PS exposure in CD34^+^ CB cells (Supplementary Fig. 3M). Thus, although Gal-9 is a potent inducer of PS exposure, Gal-9-induced cell death does not require caspase activation.

### Galectin-9 inhibits the execution of autophagy in AML cells

**Fig. 4:**
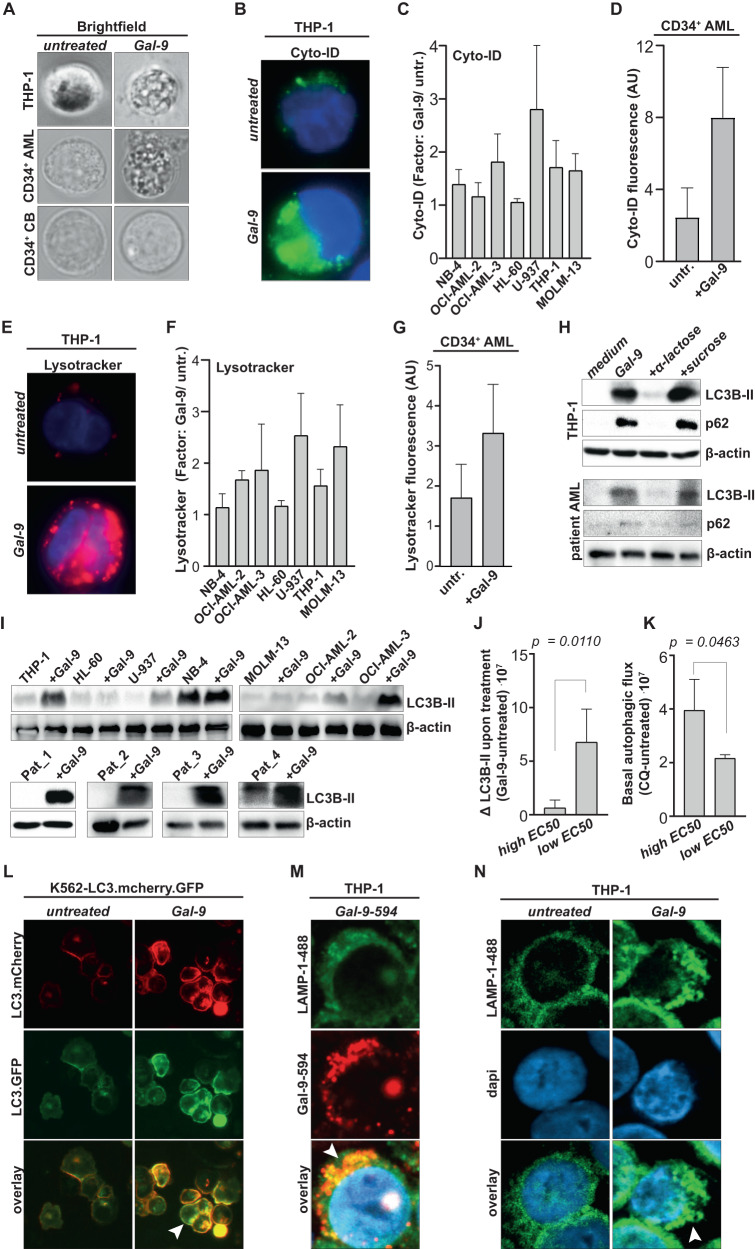
Gal-9 inhibits autophagy in AML cells. **A** Images of AML cells (THP-1 and patient-derived CD34^+^ cells) and CB-derived CD34^+^ cells upon treatment with Gal-9 (300 nM, 16 h), analyzed with fluorescent microscopy. **B** image of THP-1 cells upon treatment with Gal-9 (300 nM, 16 h) and stained with Cyto-ID, analyzed with fluorescent microscopy. **C** As in (**B**) whereby the fluorescent signal was quantified using ImageJ for the AML cell line panel (*n* = 3). **D** as in (**C**) but using CD34^+^ patient-derived AML cells (*n* = 5). **E** Image of THP-1 cells upon treatment with Gal-9 (300 nM, 16 h) and stained with Lysotracker, analyzed with fluorescent microscopy. **F** As in (**E**), whereby the fluorescent signal was quantified using ImageJ for the AML cell line panel (*n* = 3). **G** as in (**F**) but using CD34^+^ patient-derived AML cells (*n* = 5). **H** Western blot of autophagy-related proteins SQSTM1/p62 (62 kDa) and LC3B-II (16 kDa), including loading control beta-actin (42 kDa), upon incubation with Gal-9 (300 nM, 6 h) in the THP-1 cell line and a patient-derived CD34^+^ AML sample. **I** Western blot of the whole cell line panel as well as four different patient-derived AML samples detecting LC3B-II (16 kDa) and the loading control beta-actin (42 kDa) upon treatment with Gal-9 (300 nM, 16 h). **J** Association of the Gal-9 sensitivity (either low EC50/highly sensitive or high EC50/weakly sensitive) with the accumulation of LC3B-II upon treatment with Gal-9, as shown in (**I**). **K** As in (**J**), but comparing sensitivity for Gal-9 with the basal autophagic flux as determined by incubation with CQ (Supplementary Fig. 4B). **L** Fluorescent images of the K562-LC3.mCherry.GFP model cell line treated with Gal-9 (150 nM, 16 h). The arrow highlights cells with mCherry-GFP double-positive cells. **M** Representative confocal images of Gal-9 accumulation in the lysosomes of AML cells using fluorescently labeled Gal-9 (Gal-9-594; red) and counter-stained for LAMP-1 (LAMP1-488; green) and dapi (blue). **N** As in (**M**), focusing on the lysosomal morphology upon treatment with Gal-9. The arrow highlights swollen lysosomes.

In colon carcinoma, we identified that Gal-9-induced cell death was characterized by prominent vacuolization and depended on the autophagy pathway [14]. Analogously, Gal-9 treatment of AML cells triggered prominent vacuolization, a hallmark of autophagy, as illustrated for THP-1 and CD34^+^ AML cells, which were not detected in healthy CD34^+^ CB stem cells (Fig. 4A). These vacuoles were partly characterized as autophagosomes with a clear increase in Cyto-ID staining in both cell lines (Fig. 4B, C) and CD34^+^ patient-derived AML cells (Fig. 4D). In addition, accumulation of lysosomes was detected, whereby Gal-9 triggered a strong increase in Lysotracker staining in AML cell lines (Fig. 4E, F) and CD34^+^ patient-derived AML samples (Fig. 4G).

To further investigate the involvement of the autophagy pathway, the hallmark markers LC3B-II and p62/SQSTM1 were assessed. LC3B-II is produced during the activation of autophagy to form the autophagosome, whereas p62 is a cargo protein that helps to entrap target material in the autophagosomes [19]. During the execution phase of autophagy, when the autophagosome fuses with the lysosome, p62 and most of the LC3B-II are degraded. Therefore, persistent accumulation of LC3B-II and p62 is a sign of halted autophagy. Gal-9 induced a CRD-dependent increase in both LC3B-II and p62 levels in AML cell lines (Fig. 4H, Supplementary Fig. 4A) as well as in a patient-derived CD34^+^ AML sample (Fig. 4H) upon 6 h of incubation. This accumulation in LC3B-II was persistent in both AML cell lines and patient-derived AML cells as it retained after 16 h of incubation (Fig. 4I). Of note, Gal-9 did not or only minimally induce LC3B-II accumulation in CB cells, analogously to the insensitivity of these cells to Gal-9 (Supplementary Fig. 4C). The extend of LC3B-II accumulation was not equal in all AML cell lines and associated with the sensitivity of the cell line for Gal-9. Specifically, the accumulation in LC3B-II was significantly stronger in cell lines with a higher sensitivity for Gal-9 (and thus a lower EC50) compared to cell lines that were less sensitive (Fig. 4J and Supplementary Fig. 4B, D). Furthermore, the basal level of autophagic flux, as identified by accumulation of LC3B-II upon treatment with the lysosomotropic agent chloroquine (CQ) (Supplementary Fig. 4B), is significantly associated with sensitivity for Gal-9. Here, cell lines with a low basal autophagic flux were more sensitive for Gal-9 (Fig. 4K and Supplementary Fig. 4E). The endogenous expression of Gal-9 did not associate with sensitivity to treatment with exogenous Gal-9 in either cell lines or patient-derived AML samples (Supplementary Fig. 4F, G).

The above-described data indicate that Gal-9 disturbs the proper execution of autophagy at the stage of autophagolysosome formation. This was confirmed in the K562 cell line (that is equally sensitive to Gal-9 as the AML cell line panel, Supplementary Fig. 4H), which was transduced with the LC3.mCherry-GFP construct. Here, the formation of autophagolysomes will yield mCherry-positive vesicles, whereas the inhibition of autophagy results in mCherry.GFP double positive vesicles. Indeed, K562-LC3.mCherry.GFP cells treated with Gal-9 accumulated mCherry-GFP double positive vesicles (Fig. 4L), similar to the autophagy inhibitor CQ (Supplementary Fig. 4I). In contrast, the positive control ‘starvation’ did induce autophagolysosomes with diminished GFP-signal (Supplementary Fig. 4I). Halted autophagy may stem from impaired lysosomal functioning. Indeed, Gal-9 accumulated in the lysosomes of AML cells (Fig. 4M) and induced lysosomal swelling (Fig. 4N), suggesting that Gal-9 acts as a lysosomotropic agent.

**Fig. 5:**
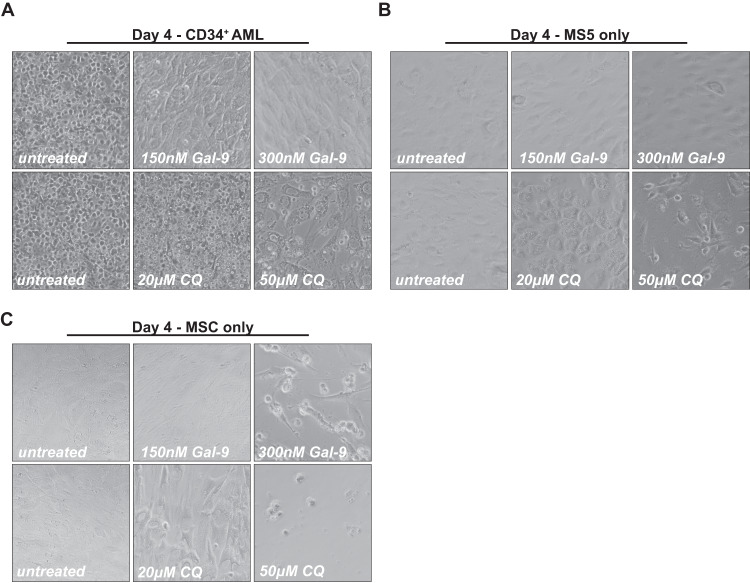
Gal-9 is more potent to eliminate AML with less toxicity towards stromal cells compared to CQ. **A** Pictures of CD34^+^ patient-derived AML cells treated with Gal-9 (150, 300 nM) and CQ (20, 50 µM) for 4 days on top of an MS5 monolayer (light microscopy). **B** Image of MS5 cells only, treated with the indicated concentrations of Gal-9 and CQ for 4 days (light microscopy). **C** As in (**B**) but using primary human mesenchymal stem cells (MSC).

Recent studies highlight that targeting the lysosomes may be a potential novel therapeutic strategy to treat AML [20–23], with CQ being commonly the agent of choice. Therefore, the cytotoxic potential of CQ and Gal-9 was compared, with 150 nM and 300 nM Gal-9 and 20 µM and 50 µM CQ (Supplementary Fig. 5A, B) being defined as optimal concentrations. In patient-derived AML cells, treatment with 150 nM Gal-9 was sufficient to achieve 90% cell death after 16 h and complete removal after 4 days of treatment. In contrast, 50 µM CQ reduced the viability by only 40%, reaching up to 80% after 4 days of treatment (Fig. 5A, Supplementary Fig. 5C). Importantly, whereas the MS5 stromal layer underneath Gal-9-treated AML cells remained healthy, this layer was strongly affected upon CQ-treatment (Fig. 5A). Indeed, Gal-9 did not negatively impact on the cell viability of MS5 in a single culture, whereas the effective dose of CQ for AML cell killing (50 µM) had a clear negative impact on MS5 cells (Fig. 5B). Gal-9 did also not affect human mesenchymal stem cells (MSC) at 150 nM, whereas CQ at 20 µM already significantly reduced viability (Fig. 5C). Furthermore, the MSC monolayer was completely eradicated when treated with 50 µM CQ, whereas, although clearly affected, a substantial amount of MSCs remained upon treatment with 300 nM Gal-9 (Fig. 5C).

Taken together, Gal-9 inhibited the execution of autophagy by accumulating in the lysosomes, inducing swelling of these organelles, and thus seems to act as a lysosomotropic agent like CQ. However, although both agents induce AML cell death, Gal-9 seemed to be less toxic for stromal cells than CQ and, therefore, may be more suitable for the treatment of AML.

### Gal-9 is cytotoxic for AraC-resistant AML and can be combined with Azacitidine

**Fig. 6:**
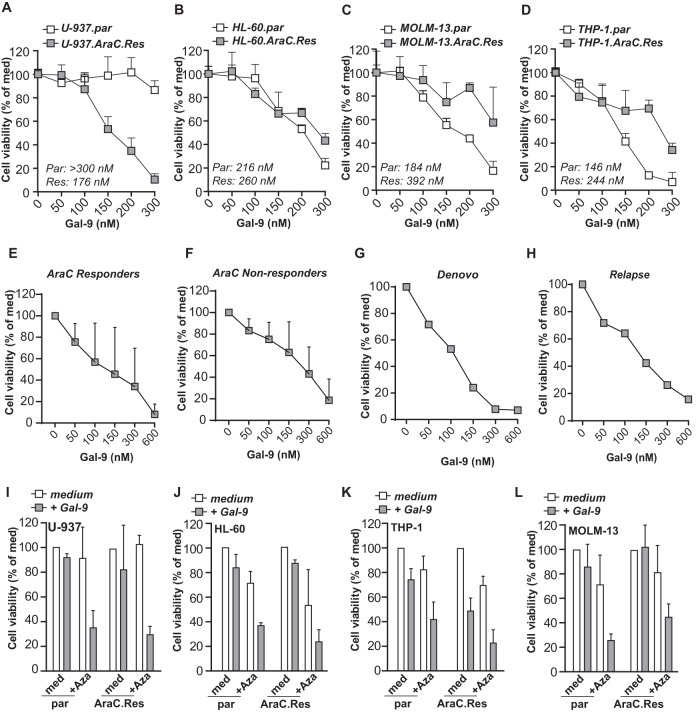
Gal-9 is cytotoxic for AraC-resistant AML and potentiates the efficacy of Aza. Cell viability as determined by the MTS assay of parental vs. AraC resistant (AraC-Res) AML cell line pairs **A** U-937, **B** HL-60, **C** MOLM-13, and **D** THP-1, treated with the indicated concentration Gal-9 for 72 h (*n* = 4). The concentrations depicted inside the graph indicate the EC50 for Gal-9. Impact of Gal-9 as determined by flow cytometry-based cell viability (Annexin V/PI) on **E** AraC-responders (*n* = 4) vs. **F** AraC-non responders (*n* = 4) after 16 h of incubation. **G** as in (**E**) and (**F**), but for a matched sample during initial disease (de novo; **G**) or relapse (**H**). Cell viability (using MTS assay) of the parental vs. AraC-resistant AML cell line panel, including **I** U-937, **J** HL-60, **K** THP-1, and **L** MOLM-13, treated with a combination of low dose Aza (2,5 µM) and Gal-9 (150 nM) (all *n* = 3). Of note, Aza was pre-incubated for 16 h before adding Gal-9 and incubating for an additional 72 h.

AraC-resistant (AraC-Res) AMLs often have defects in the apoptosis pathway [20]. Furthermore, lysosomes are known to contribute to therapy resistance by drug sequestration [21]. Since Gal-9 induces apoptosis-independent cell death and acts as a lysosomal inhibitor, we hypothesized that Gal-9 might retain cytotoxicity against AraC-Res AML cells. Indeed, Gal-9 eliminated all 4 AraC-Res AML cell lines in a dose-dependent manner (Fig. 6A–D). The EC50 in HL-60 cells did not differ between parental and AraC-Res cells, slightly increased in AraC-Res MOLM-13 and THP-1 cells, but strongly reduced in U-937 cells (Fig. 6A–D). Gal-9 also increased the amount of cell death when combined with AraC compared to either agent alone in parental U-937 cells (Supplementary Fig. 5D). Furthermore, patient-derived AML cells that did not respond to AraC were eliminated by Gal-9 in a dose-dependent manner to a similar extent as samples that were sensitive to AraC (Fig. 6E, F and Supplementary Fig. 5E, F). Interestingly, in a unique matched de novo and relapsed patient sample pair, Gal-9 efficiently killed patient-derived AML cells during both stages of the disease (Fig. 6G, H).

Interestingly, an increase in cell death was observed when Aza was combined with Gal-9 compared to either agent alone in parental (Fig. 6I–L) and AraC-resistant AML cell lines. In contrast, the combination of Aza and AraC was only effective in parental cells (Supplementary Fig. 5G–J). Thus, the addition of Gal-9 to Aza therapy may be of interest to achieve higher response rates in AML patients that are ineligible for AraC treatment or patients that relapsed after initial AraC therapy.

## Discussion

In the current study, we identified that Gal-9 induced caspase-independent cell death in AML cell lines and patient-derived AML cells yet did not affect healthy CB-derived CD34^+^ stem cells. This induction of cell death was associated with the accumulation of Gal-9 in lysosomes inducing their swelling and inhibiting the execution phase of autophagy. Importantly, Gal-9 cytotoxicity was retained in AraC-resistant AML cells and could be used in combination with Aza. This sensitivity of AraC-resistant AMLs to Gal-9 cytotoxicity is of potential clinical relevance since new treatment modalities that could surmount apoptosis resistance in AML cells are urgently needed.

As demonstrated in this study, treatment with Gal-9 resulted in halted autophagy and concomitant cytotoxic elimination of AML cells. This finding is similar to our previous study in colorectal cancer [14], which implies that Gal-9 has a unique cytotoxic potential with a common mechanism of action in different cancer types. Autophagy is an essential catabolic process that has been associated with survival and homeostasis in cancer. For instance, increased levels of autophagy were reported in resistant AML cells upon treatment with AraC [22–26] and other drugs like hDAC and mTOR inhibitors [27, 28]. Therefore, agents that inhibit autophagy have been explored for the treatment of AML, which uniformly demonstrated that autophagy inhibition during initial treatment increases treatment efficacy [22–26]. However, timing is an important issue since we recently demonstrated that AML cells that already gained resistance to AraC could not be re-sensitized with autophagy inhibitors [24]. Interestingly, in the current study, both AraC-sensitive and AraC-resistant AML cells were Gal-9 sensitive. This suggests that besides autophagy inhibition, Gal-9 may have additional working mechanisms not yet elucidated in AML. Potential mechanisms of action are the induction of reactive oxygen species or via the calcium calpain pathway, as has been respectively demonstrated in ovarium carcinoma [18] and T cell leukemia [29]. Interestingly, endogenous Gal-9 has been reported to regulate autophagy by interacting with lysosome-associated membrane protein 2 (LAMP-2) and, hence, was found to be enriched in the lysosomes [30]. Correspondingly, we observed that exogenously added Gal-9 accumulated in the lysosomes of AML cells leading to lysosomal swelling, which was in line with our previous study [14]. Whether this accumulation is also the result of Gal-9 interacting with LAMP-2 is still the subject of investigation, but the accumulation of Gal-9 in lysosomes suggests that Gal-9 acts as a lysosomotropic agent.

In AML and solid malignancies, lysosomes are known to sequester chemotherapeutic agents, thereby impacting their therapeutic efficiency and governing resistance [31]. Therefore, the use of lysosomotropic agents has been proposed to increase treatment efficacy during chemotherapy, with CQ being the most commonly used agent [32–35]. However, the clinical use of CQ or its derivative hydroxychloroquine is hampered by dose-limiting toxicities, inducing, among others, hypoglycemia and bone marrow suppression at doses not impacting the cancer cells [35]. In line with this data, CQ was cytotoxic for both the stromal cell line MS5 and human MSCs at a dose that only affected ~50% of the patient-derived AML cells in the current study. In contrast, Gal-9 did not negatively impact MS5 cells, even at the highest dose, and had a less detrimental effect on human MSCs, whilst killing the majority of the patient-derived AML cells. Furthermore, Gal-9 also seems to have a better therapeutic window in stem cells than CQ, as CB-derived healthy CD34^+^ stem cells were equally sensitive to treatment with CQ as AML stem cells [36], whereas Gal-9 did not have any negative impact on CB-derived healthy CD34^+^ stem cells in the current study. Of note, Gal-9 also reduced the percentage of CD34^+^ patient-derived AML cells, although there was no significant difference in sensitivity between CD34^+^ and CD34^−^ cells. This suggests that Gal-9 may induce differentiation of AML stem cells, which is in line with its potential to drive differentiation of various other cell types, among which are immune cells [37–39] and osteoblasts [40]. Furthermore, Gal-9 also did not have any toxic effects on healthy melanocytes or colon epithelial cells in our previous studies [13, 14]. Therefore, Gal-9 may be a better option than CQ to block lysosomal functioning, having fewer cytotoxic side effects towards healthy stromal cells, CD34^+^ stem cells, and epithelial cells. The reason for the cancer-specific cytotoxic effect of Gal-9 is currently unclear. First, it may be caused by differences in the dependency of the autophagy pathway between healthy and malignant cells, as cancer cells commonly rely on the autophagy pathway to survive [36]. Second, cancer cells commonly harbor a differential glycosylation pattern compared to healthy cells [41], which may cause increased binding of Gal-9 towards malignant cells.

Despite the potent anticancer activity of Gal-9 and seemingly low impact on healthy cells, it is important to take the pleiotropic activity of this lectin towards immune cells into account. On the one hand, Gal-9 is capable of activating immune cells and, thereby, eliciting an anti-cancer immune response. Indeed, we demonstrated that Gal-9 activates neutrophils, resulting in increased cytokine secretion, migration, and survival in vitro [42]. Furthermore, we and others delineated that a low dose of Gal-9 activates T cells and triggers their expansion [43, 44], which, although not formally proven yet, may drive anti-cancer immune responses. On the other hand, Gal-9 is known to induce apoptosis at higher concentrations in T-helper 1 and T-helper 17 cells [45], and its expression is associated with T-cell effector dysfunction in the tumor microenvironment [46]. Furthermore, Gal-9 has been reported to drive self-renewal of AML stem cells and leukemic progression [46], although this effect was observed at a concentration of 500 pg/ml Gal-9, which is 2 × 10^4^ times lower than the 300 nM we used in our study and that eliminated the fast-majority of CD34^+^ AML stem cells. However, the negative impact of Gal-9 on T cells is a potential limitation in treating patients with Gal-9. One possibility to safely incorporate Gal-9 treatment into clinical practice would be during hematopoietic stem cell transplantation [47]. During this therapy, AML patients receive a high dose of chemotherapy to eradicate all malignant cells. At this stage, Gal-9 can be administered at a high dose, whereby the negative impact on T cells would be of no consideration, as they will be replenished after transplantation. Furthermore, as we demonstrated, Gal-9 can be used in combination with Aza and AraC, which suggests that Gal-9 can be added to salvage therapy prior to stem cell transplantation.

In conclusion, Gal-9 has potent cytotoxic activity toward AML cells but not toward healthy CB-derived cells and stromal cells, which is not hampered by AraC resistance. Therefore, Gal-9 may be a potential novel therapeutic agent for AML patients in general, as well as patients with AraC-resistant relapses.

## Materials and methods

### Cell lines and galectin-9

THP-1, HL-60, U-937, MOLM-13, NB4, OCI AML2, OCI AML3, K562, and MS5 were originally obtained from American Type Culture Collection (ATCC). All cell lines were cultured at 37 °C in a humidified 5% CO_2_ atmosphere in RPMI (Lonza 12–115F, Basel, Switzerland) supplemented with 10% fetal calf serum (FCS) (Sigma Aldrich, F7524, St. Louis, MO, USA) and regularly tested for mycoplasma. Cytarabine-resistant cell lines were generated as described previously by us [24]. The K562.LC3.mCherry-GFP cell line was generated previously by our laboratory [36]. Recombinant Galectin-9 (Gal-9) was produced as previously described [48]. AraC and Aza were from the UMCG hospital pharmacy.

### Ex vivo culturing of patient-derived AML and cord-blood cells

Patient-derived AML samples (stored after informed consent and following approval by the Medical Ethical Committee of the UMCG in accordance with the Declaration of Helsinki protocol code NL43844.042.13, 6 January 2014) were thawed from cryovials and added to pre-warmed newborn calf serum (NCS, Gibco, Breda, The Netherlands), and centrifuged at 450*g* for 5 min. The cell pellet was then resuspended in pre-warmed NCS mix (5 U/mL Heparin (Pharmacy of the UMCG), 4 µM magnesium sulfate (Sigma-Aldrich, St. Louis, MO, USA), and 20 U/mL DNase (Roche, Basel, Switzerland)) and incubated for 15 min in a 37 °C water bath. Thereafter, AML cells were washed and cultured in Gartner’s medium (Alpha-MEM, 12.5% horse serum, 12.5% FCS, 1 µM hydrocortisone (Sigma-Aldrich, St. Louis, MO, USA), 1% pen-strep (Sigma-Aldrich, St. Louis, MO, USA), and 50 µM beta-mercaptoethanol (Sigma-Aldrich, St. Louis, MO, USA)) supplemented with thrombopoietin (TPO) and G-CSF, IL-3 (20 ng/mL of each cytokine) (hospital pharmacy, UMCG), as described before [49] on top of an MS5 support-layer grown on gelatin-coated flasks for 2–3 days. After this recovery period, primary samples with the viability of over 80% were used in cell death and autophagy assays. Primary human mesenchymal stem cells (MSCs) were cultured on gelatin-coated flasks for 2–3 days in Alpha-MEM (Sigma-Aldrich, St. Louis, MO, USA) supplemented with 1% pen-strep (Sigma-Aldrich, St. Louis, MO, USA), 10 U/mL Heparin (Pharmacy of the UMCG) and 5% platelet lysate (Sigma-Aldricht, St. Louis, MO, USA).

Mononuclear cells (MNCs) were isolated from the CB of healthy subjects and separated using ficoll (Lymphoprep™, Bernburg, Germany), after which CD34^+^ stem cells were isolated by MACS sorting using CD34 MACS microbeads (Miltenyi Biotec, Leiden, The Netherlands) following manufacturers recommendations. CD34^-^ cells. CD34^+^ from patient-derived AML samples were isolated in the same way.

### Cytotoxicity assays

To determine the cytotoxicity induced by AraC, Aza, Gal-9, and CQ (from the LC3B Antibody Kit for Autophagy, L10382, Invitrogen^TM^, Carlsbad, CA, USA), 5 × 10^4^ cells (either cell lines or primary patient-derived material) were plated in a 48 well plate with 200 µL RPMI supplemented with 10% FCS (for cell lines) or Gartner’s medium (for patient-derived AML cells or CB cells) and treated with the indicated concentrations of Gal-9, AraC, Aza or CQ. In the case of combinational treatments, Gal-9 and AraC were added simultaneously, whereas cells were pre-incubated with Aza for 16 h before adding Gal-9. For experiments with Z-VAD-fmk (R&D Systems, Inc., FMK001, Wiesbaden, Germany), cells were pre-treated with 20 µM Z-VAD-fmk for 16 h and again treated with 20 µM Z-VAD-fmk freshly added 1 h before adding Gal-9 or Staurosporine (Sigma Aldrich, St. Louis, MO, USA). For long-term assays using patient-derived AML cells (5–7 days), wells were first coated with MS5 cells (pre-plated 1 day before the assay to reach confluency at the day of use) before AML cells were layered on top of the stromal layer. For the CQ assays, cells were incubated on top of MS5 cells. Cytotoxicity was assessed using either flow cytometry-based cell counts, Annexin-V staining, DioC6 staining, or MTS assay.

#### Flow cytometry-based cell count

After 24 h of incubation, cells were harvested and counted using a flow cytometer (BD Accuri^TM^ C6 cytometer, BD Biosciences, San Jose, CA, USA or Cytoflex, Beckman Coulter, Brea, CA, USA, and accessory software). Here, cells/µl in the ‘viable’ gate were determined (Fig. 1B).

#### MTS assay

After 72 h of incubation, cell viability was assessed using the MTS assay (CellTiter 96^®^ AQueous One Solution Cell Proliferation, G3580, Promega, Madison, WI, USA). In brief, MTS was added (7.5% v/v) to each well and incubated at 37 °C. After sufficient color development, read-out was performed at OD490nm (Multi-Scan Sky of Thermo Scientific Waltham, MA, USA). Cell viability was calculated by subtracting OD490nm of the dead control of each value (7.5% ‘dead mix’, consisting of 10% Triton-X in 70% ethanol) and calculating the viability as % of the untreated control (treated/untreated*100%).

#### Annexin-V assay

After 24 h of incubation, cells were harvested and stained with Annexin-V. In brief, cells were resuspended in 1x Annexin V binding buffer (10x Annexin V binding buffer, BD Biosciences, San Jose, CA, USA), and 1% Annexin-V-FITC (Immunotools, Friesoythe, Germany) was added. After 10 min incubation at 4 °C, staining was analyzed using flow cytometry.

#### DioC6 staining

DiOC6 (Molecular Probes, Eugene, Oregon, USA) at a concentration of 0.1 µM in fresh culture medium was added 1:1 to cells. After 20 min of incubation at 37 °C, cells were washed with PBS, and collected by centrifugation (450 g, 5 min), resuspended in PBS, and analyzed using flow cytometry.

To determine the percentage of CD34^+^ AML cells after treatment with Gal-9, AML cells were pre-incubated with FcR-blocker (100μg/mL) (Miltenyi Biotec, Leiden, The Netherlands) and subsequently stained with anti-CD34 antibody ((Clone: 561, Biolegend, California, USA) for 1 h at 4^o^C. Cells were washed with PBS and collected by centrifugation (450*g*, 5 min), resuspended in PBS, and analyzed using flow cytometry.

### Autophagosomal and lysosomal content: Cyto-ID and lysotracker

To determine the autophagosomal and lysosomal content in cell lines and AML patient-derived samples, cells were cultured at a density of 5 × 10^4^ in a 48 wells plate and treated with Gal-9 (300 nM) or with CQ (10 µM) for 16 h. Subsequently, LysoTracker® Red DND-99 (1 μM, Life Technologies, L-7528, Carlsbad, California, USA) or cyto-ID was added in combination with Hoechst (Cyto-ID autophagy detection kit; ENZ-51031–0050, Enzo Lifesciences, Inc., New York, USA) following the manufacturer’s recommendation, whereupon cells were incubated for 30 min at 37 °C. Dye excess was washed away with PBS, and staining was visualized using IncuCyte S3 Live-Cell Analysis System (Ann Arbor, MI, USA) or the EVOS Cell Imaging System (EVOS-FL, Thermo Scientific, Waltham, MA, USA). The corrected total cell fluorescence (CTCF) of LysoTracker and Cyto-ID signal was determined using ImageJ software.

### Western blot analysis of autophagic flux and caspase-3 activation

To detect the basal level of autophagic flux in AML and CB cells, 1 × 10^6^ cells were cultured in a 6-well plate and treated with CQ (50 µM) for 6 h or 24 h. To determine the impact of Gal-9 on the autophagy pathway, cells were treated with Gal-9 (300 nM) for 6 h or 24 h. Cell lysates were prepared using lysis buffer (50 mM Tris, 2 mM EDTA, 2 mM EGTA, 150 mM NaCl, 0.1% SDS, 1% NP-40 substitute) containing 1 µM Na_3_VO_4_ (Sigma, 450243, St. Louis, MO, USA) and protease inhibitor cocktail (Sigmafast; Sigma Aldrich, S8820, St. Louis, MO, USA). Protein concentration was determined using Bradford protein assay (Pierce™ Coomassie (Bradford) Protein Assay Kit, #23200, Thermo Scientific, Waltham, MA, USA) then 20 µg of total protein was loaded to SDS-PAGE gels for electrophoresis using a 15% gel. Next, proteins were transferred to a nitrocellulose membrane (Amersham Hybond ECL; GE Healthcare, RPN303D, Merck, Darmstadt, Germany). After blocking in 5% (w/v) milk powder/TBST, proteins were detected using primary antibodies against LC3B (LC3B Antibody Kit for Autophagy, Invitrogen^TM^, L10382, Carlsbad, CA, USA), SQSTM1/ p62 (SantaCruz, sc-28359, California, USA), β-actin (Abcam, ab49900, Cambridge, UK), total caspase 3 (#9665 cell signaling, Danvers, Massachusetts, USA), cleaved caspase 3 (#9661 cell signaling, Danvers, Massachusetts, USA), and appropriate secondary HRP-conjugated antibodies (Dako, p0217, p0260, Santa Clara, USA). Blots were developed using chemiluminescent substrate (SuperSignal West Dura, Thermo Scientific, Life Technologies, 34075, Waltham, MA, USA) and imaged using the ChemiDoc MP system (Bio-Rad, Hercules, California, USA). Quantification of detected protein levels was performed using the ImageJ tool for gel analysis.

### Confocal microscopy

To detect Gal-9 accumulation in lysosomes as well as lysosomal swelling upon Gal-9 treatment, THP-1 cells were seeded in a 48 wells plate (5 × 10^5^ cells/mL) and treated with Gal-9-Alexa-594 overnight. Gal-9-Alexa-594 was prepared using DyLight® 594 (DyLight 594 NHS Ester; Piercenet, Thermo Scientific, 46412, Waltham, MA, USA) following the manufacturer’s protocol. Subsequently, cells were transferred to a microscope slide using a cytocentrifuge (Cytospin 3, Shandon, England). 4% w/v paraformaldehyde (PFA) was used for fixation for 15 min, and then the lysosomes were stained with anti-LAMP-1-488 (sc-20011 AF488 Santacruz Biotechnology, Dallas, Texas, USA) in a culture medium containing 40 mM α-lactose to prevent any direct interaction of Gal-9 with the LAMP-1 antibody. After 1 h incubation at RT, the cells were washed twice with TBST, and nuclei were stained with DAPI in a mounting medium (Sigma Aldrich, D9542, St. Louis, MO, USA) and subsequently visualized using a Leica SP8 Confocal microscope (Leica Microsystems, Rijswijk, The Netherlands). To determine the autophagic flux using K562 cells transfected with the LC3-mCherry-GFP construct, cells were seeded in a 48 wells plate (5 × 10^5^ cells/mL) and overnight treated with Gal-9 (150 nM), CQ (50 µM) or starvation (serum-free RPMI). Subsequently, cells were spotted on a microscope slide, and images were made without prior fixation (as this can recover the GFP signal due to neutralization of the pH [50]).

### RTqPCR

mRNA was isolated from AML cell lines as well as patient-derived AML cells using the mRNA isolation kit (Qiagen RNeasy plus mini kit #74134). Subsequently, mRNA was converted into cDNA using the iScript™ cDNA Synthesis Kit (Biorad; #1708891). For the RTqPCR reaction, SYBRgreen was used (Biorad, #1725274), applying 5 ng cDNA per condition and using the following primers; *LGALS9* (forward: GCTGTGAACTTTCAGACTGGC, reverse: AGCAGAGGTCAAAGGGCATC and *RPL27 (*forward: TCCGGACGCAAAGCTGTCATCG*,* reverse: TCTTGCCCATGGCAGCTGTCAC). RTqPCR reaction was performed using the thermocycler (Biorad C1000, CFX384 Real-Time System) qPCR program: 3 min 95 °C, (5 s 95 °C, 15 s 58 °C → 39 times), 3 s 65 °C, 5 s 95 °C.

### Statistical analysis

EC50 and sigmoidal curve fitting correlation coefficients of Gal-9 were calculated using the ED50 Plus v1.0 Excel worksheet developed by Dr. Mario H. Vargas at Instituto Nacional de Enfermedades Respiratorias. For each experiment, the sample size ‘*n*’ is stated in the figure or in the figure legend, and all graphs depict the mean ± standard deviation. Significance was tested using Wilcoxon signed-rank test (treated vs. untreated in the same sample) or, in case the samples were not ranked (different samples in the groups), using the Mann-Whitney U test using GraphPad Prism software (GraphPad Prism; GraphPad Software, La Jolla, CA, USA). The sample size to ensure significant power was based on our previously published effects of Gal-9 [14]. *p* values are indicated as: ****p < 0.0001, ***p < 0.001, **p < 0.01, and *p < 0.05.

## Supplementary information

Suppl legends

Suppl. Figure 1

Suppl. Figure 2

Suppl. Figure 3

Suppl. Figure 4

Suppl. Figure 5

Suppl. Table 1

Uncropped western blots

## Data Availability

All data are available in the main text or the supplementary materials.
