# Supplementary material for: Galectin-9 has non-apoptotic cytotoxic activity toward acute myeloid leukemia independent of cytarabine resistance
Source: Cell Death Discov. 2023 Jul 6;9:228. doi: 10.1038/s41420-023-01515-w (PMC10322858; doi:10.1038/s41420-023-01515-w)
Supplement: Supplementary file 1 — Suppl legends [file 41420_2023_1515_MOESM1_ESM.docx]

**Suppl. Figure 1: Gal-9 is cytotoxic for AML cell lines.**

(**A-E**) Flow cytometry-based cell counts for the different AML cell lines upon treatment with the indicated concentrations of Gal-9 (16h incubation, n=5). (**F-J**) Cell viability as determined by the MTS assay for the different AML cell lines upon treatment with the indicated concentrations of Gal-9 (16h incubation, n=10). (**K**) As in (**A-E**), but in the presence of α-lactose (40mM) or sucrose (40mM) using 300nM Gal-9 (n=5). (**L**) As in (**F-J**), but in the presence of α-lactose (40mM) or sucrose (40mM) using 300nM Gal-9 (n=5). (**M**) EC50 for Gal-9 as determined based on the concentration curves as depicted in (**A-J**).

**Suppl. Figure 2: Gal-9 is cytotoxic for patient-derived AML cells.**

(**A**) Microscopic pictures of CD34^+^ patient-derived AML cells treated with Gal-9 (300nM) in liquid culture or on top of a MS5 support layer in the presence or absence of the CRD-blocking sugar α-lactose (40mM). (**B**) Analysis to determine the difference in sensitivity of CD34^+^ vs. CD34^-^ patient-derived AML cells for Gal-9 (300nM) treatment in short-term (16h) liquid culture (cell count based, both groups n=9). (**C**) Flow cytometry histogram of untreated (medium) vs. Gal-9-treated (50nM) patient-derived AML cells stained for the stem cell marker CD34 after 3 days of incubation, and (**D**) quantification hereof. (**E**) Representative bright field microscopy pictures of Gal-9 treated MS5 cells. Flow cytometry-based cell counts upon short-term treatment (16h) with Gal-9 (300nM) of (**F**) CD34^+^ (n=8) vs. CD34^-^ (n=7) patient-derived AML cells in MS5 co-cultures, (**G**) CD34^+^ (n=18) and CD34^-^ (n=15) patient-derived AML cells in liquid vs. MS5 co-culture, and (**H**) CD34^+^ patient-derived AML samples in short term liquid or MS5 co-cultures in the presence of the CRD-blocking sugar α-lactose (40mM). (**I**) Flow cytometry-based cell counts of long-term incubated healthy cord-blood (CB)-derived CD34^+^ cells (n=6) vs. patient-derived CD34^+^ AML cells (n=5) treated with different concentrations of Gal-9. (**J**) Flow cytometry histogram of CB-derived CD34^+^ cells vs. patient-derived CD34^+^ AML cells stained with DioC6 upon treatment with Gal-9 (300nM, 72h). (**K**) Flow cytometry-based cell counts of patient-derived CD34^-^ AML cells treated with a dose range of Gal-9 for long term culture on top of MS5 support cells (5-7 days). (**L**) Flow cytometry-based cell counts of a patient-derived CD34^+^ AML sample after the indicated days of incubation, whereby the cells were treated with Gal-9 every 3 days. Cell count of CD34^+^ patient-derived AML cells after repeated treatment with Gal-9 every 3 days for 14 days.

**Suppl. Figure 3: PS-exposure upon treatment with Gal-9 in AML.**

(**A-G**) Detection of PS-exposure on a panel of AML cell lines using flow cytometry-based Annexin-V staining after 16h of incubation using the indicated concentrations Gal-9 (n=5). (**H**) EC50 for Gal-9 as calculated using the data in (A-G). Detection of PS-exposure using flow cytometry-based Annexin-V staining on patient-derived (**I**) CD34^+^ or (**J**) CD34^-^ AML cells upon incubation with Gal-9 for 16h in liquid cultures. (**K, L**) As in (I, L) but upon incubation with Gal-9 for 16h in MS5 co-cultures. (**M**) Detection of PS-exposure using flow cytometry-based Annexin-V staining on patient-derived CD34^+^ AML vs. CB-derived CD34^+^ healthy stem cells.

**Suppl. Figure 4: Gal-9 inhibits autophagy in AML cells.**

(**A**) Western blot detection of LC3B-II (16 kDa) in AML cell lines treated with Gal-9 (300nM, 6h) in the presence or absence of α-lactose and sucrose (40mM), using beta-actin (42 kDa) as loading control. (**B**) Western blot detection of LC3B-II (16 kDa) upon treatment with Gal-9 (300nM) or chloroquine (CQ, 50µM) for 6h. (**C**) Western blot 3 different CB samples detecting LC3B-II (16 kDa) and the loading control beta-actin (42 kDa) upon treatment with Gal-9 (300nM, 16h). Quantification of the fold increase in (**D**) LC3B-II induced by Gal-9 and (**E**) basal autophagic flux induced by CQ in a panel of AML cell lines with different sensitivity to Gal-9 (n=3). (**F**) Endogenous Gal-9 expression determined by RTqPCR in the AML cell line panel. (**G**) as in (F) but using 3 CD34^+^ and 3 CD34^-^ patient-derived AML samples. (**H**) Cell counts (16h), cell viability (72h) and the accumulation of LC3B-II (16h) for the cell line K562 (n=5) to show it is equally sensitive to Gal-9 treatment as the panel of AML cell lines. Numbers in the graphs represent the EC50 values. (**I**) Fluorescent images of the K562-LC3.mcherry.GFP model cell line treated with CQ (50µM, 16h) or starvation (serum free RPMI, 16h). The arrow highlights cells with mcherry-GFP double positive cells for CQ and mcherry only positive cells for starvation.

**Suppl. Figure 5: Gal-9 is effective in AraC-resistant AML cells and can be combined with Aza**

(**A**) Flow cytometry-based cell counts of AML cell lines treated with a dose range of Gal-9 (n=5). (**B**) as in (A) but treating with CQ (n=5). (**C**) Flow cytometry-based cell counts of patient-derived CD34^+^ AML cells treated with Gal-9 or CQ. (**D**) Cell viability as determined by the MTS assay of U-937 cells treated with Gal-9 (300nM) in combination with a dose range of AraC upon 72h of incubation (n=5). Patient-derived AML samples were classified as (**E**) AraC responders (n=4) or (**F**) AraC non-responders (n=4) based on their sensitivity toward AraC as determined by flow cytometry-based cell viability upon 72h of incubation, being a non-responder when staying above 80% of cell viability even at the highest tested dose AraC. Cell viability (using MTS assay) of the parental vs. AraC resistant AML cell line panel using a low dose Aza (2,5µM) and a low dose AraC, being (**G**) 200nM for U-937, (**H**) 750nM for HL-60, (**I**) 20.000nM for THP-1 and (**J**) 750nM for MOLM-13. Of note, Aza was pre-incubated for 16h before adding Gal-9 and incubated for an additional 72h (n=3).

**Suppl. Table 1: Patient characteristics of the used patient-derived AML samples.** WBC: white blood cell counts in 10^9 per liter. FLT3-ITD: having a FLT-ITD mutation or not. NPM1: Having a NPM1 mutation or not. NGS: next generation sequencing to determine various mutations simultaneously as determined using the Illumina TruSight Myeloid Sequencing Panel.
