# Supplementary figures and images for: Galectin-9 has non-apoptotic cytotoxic activity toward acute myeloid leukemia independent of cytarabine resistance
Source: Cell Death Discov. 2023 Jul 6;9:228. doi: 10.1038/s41420-023-01515-w (PMC10322858; doi:10.1038/s41420-023-01515-w)

Suppl Figure 1

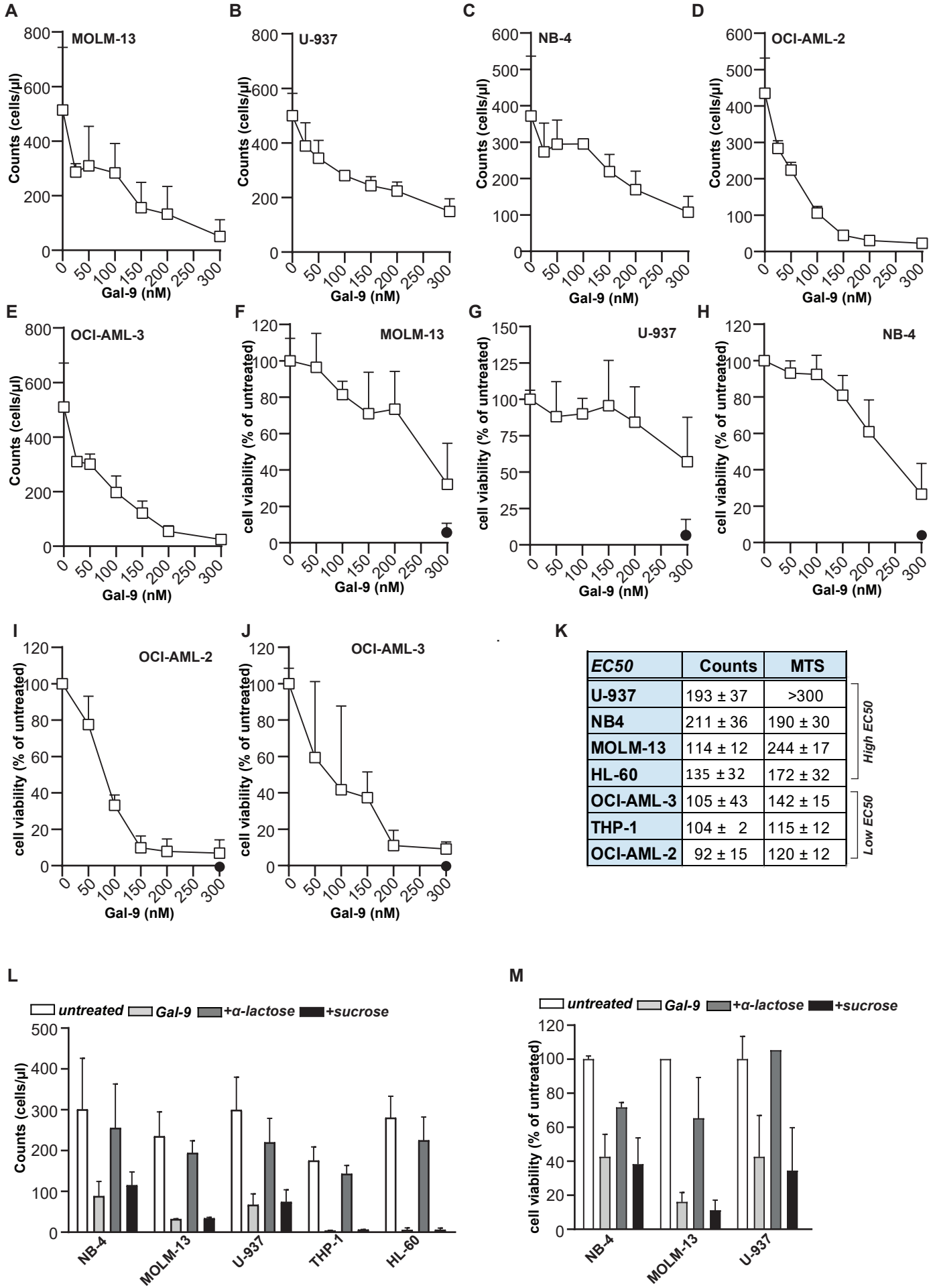

● = 600nM

Supplement: Supplementary file 2 — Suppl. Figure 1 [file 41420_2023_1515_MOESM2_ESM.pdf]

Suppl Figure 2

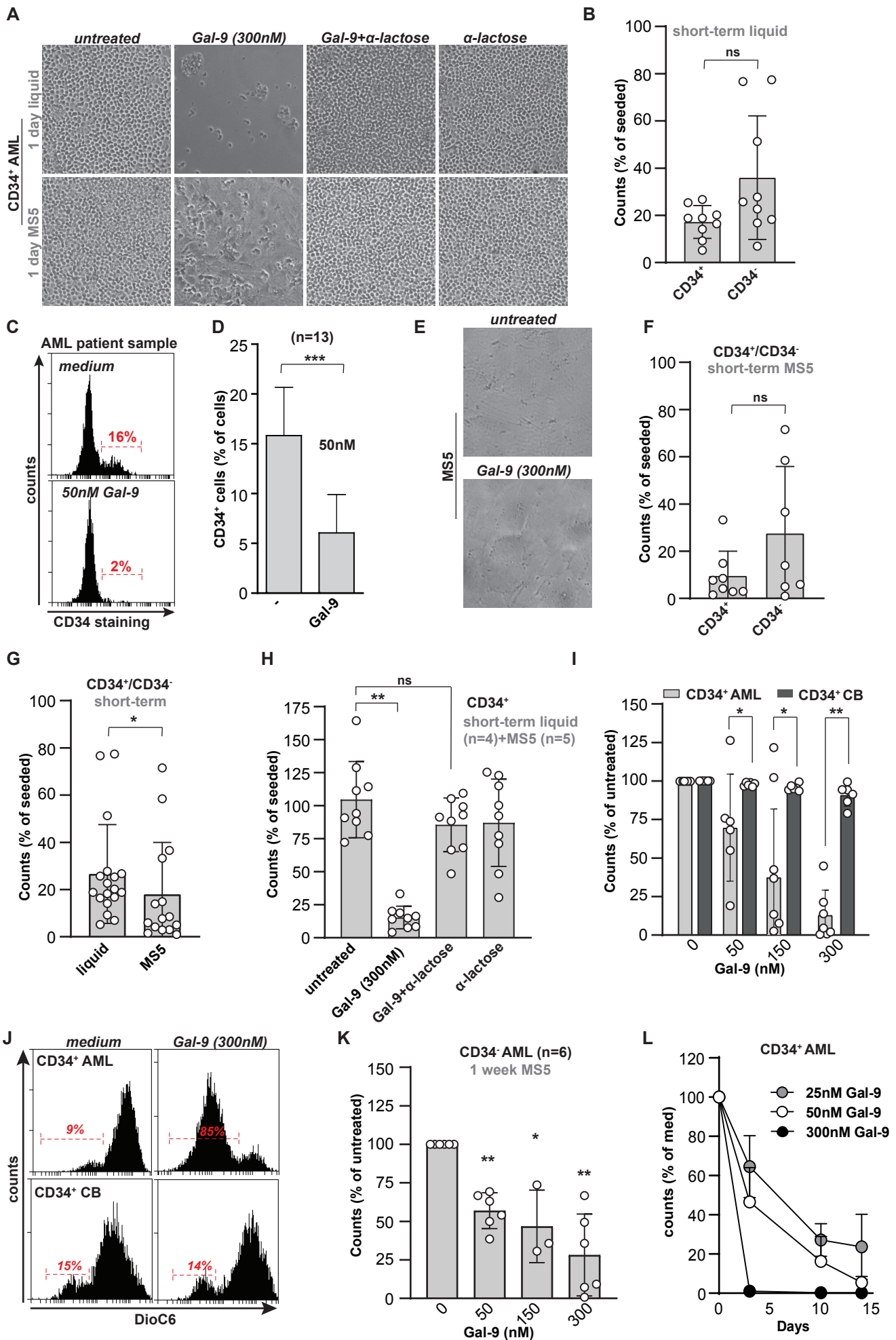

Supplement: Supplementary file 3 — Suppl. Figure 2 [file 41420_2023_1515_MOESM3_ESM.pdf]

Suppl Figure 3

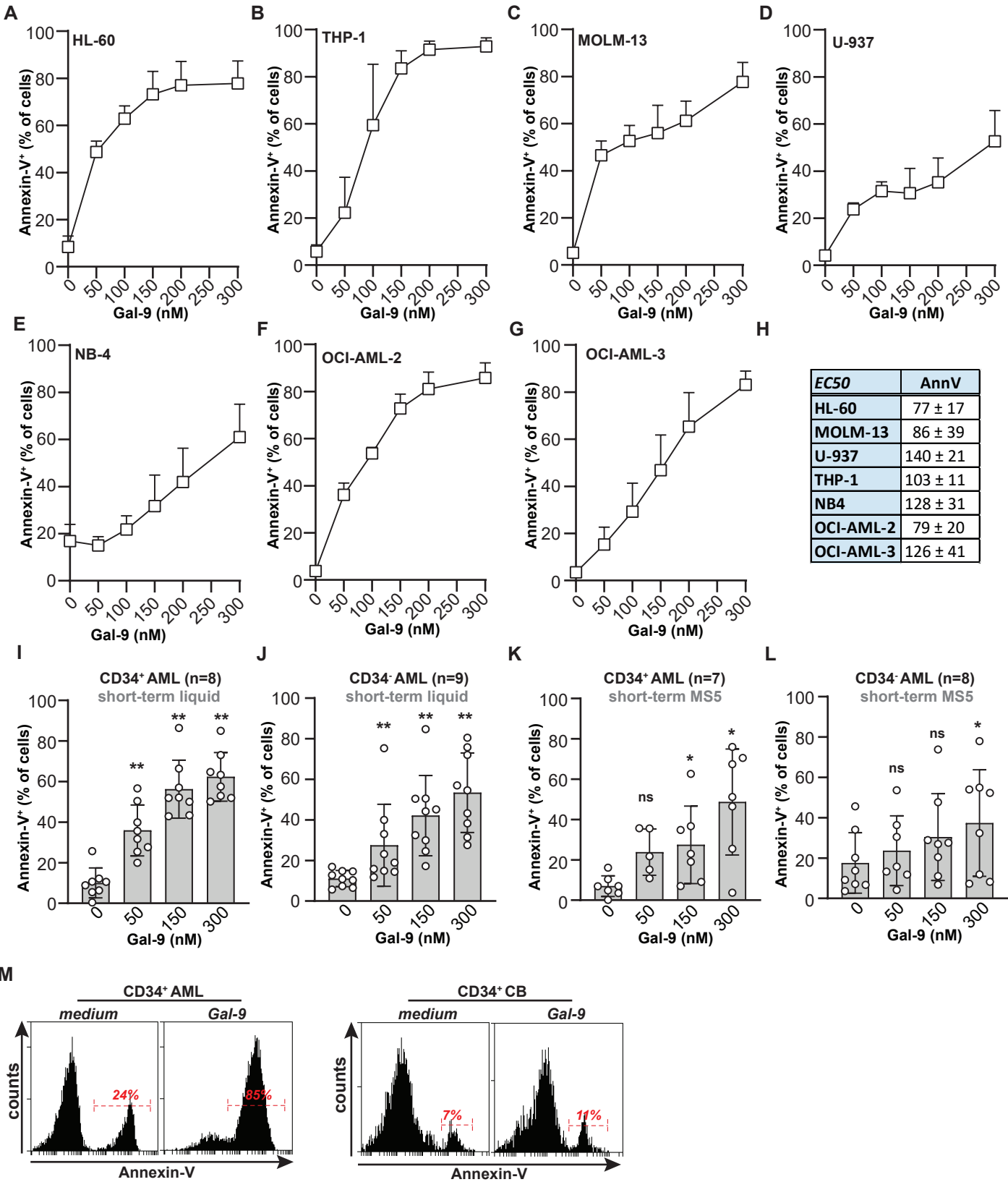

Supplement: Supplementary file 4 — Suppl. Figure 3 [file 41420_2023_1515_MOESM4_ESM.pdf]

Suppl Figure 4

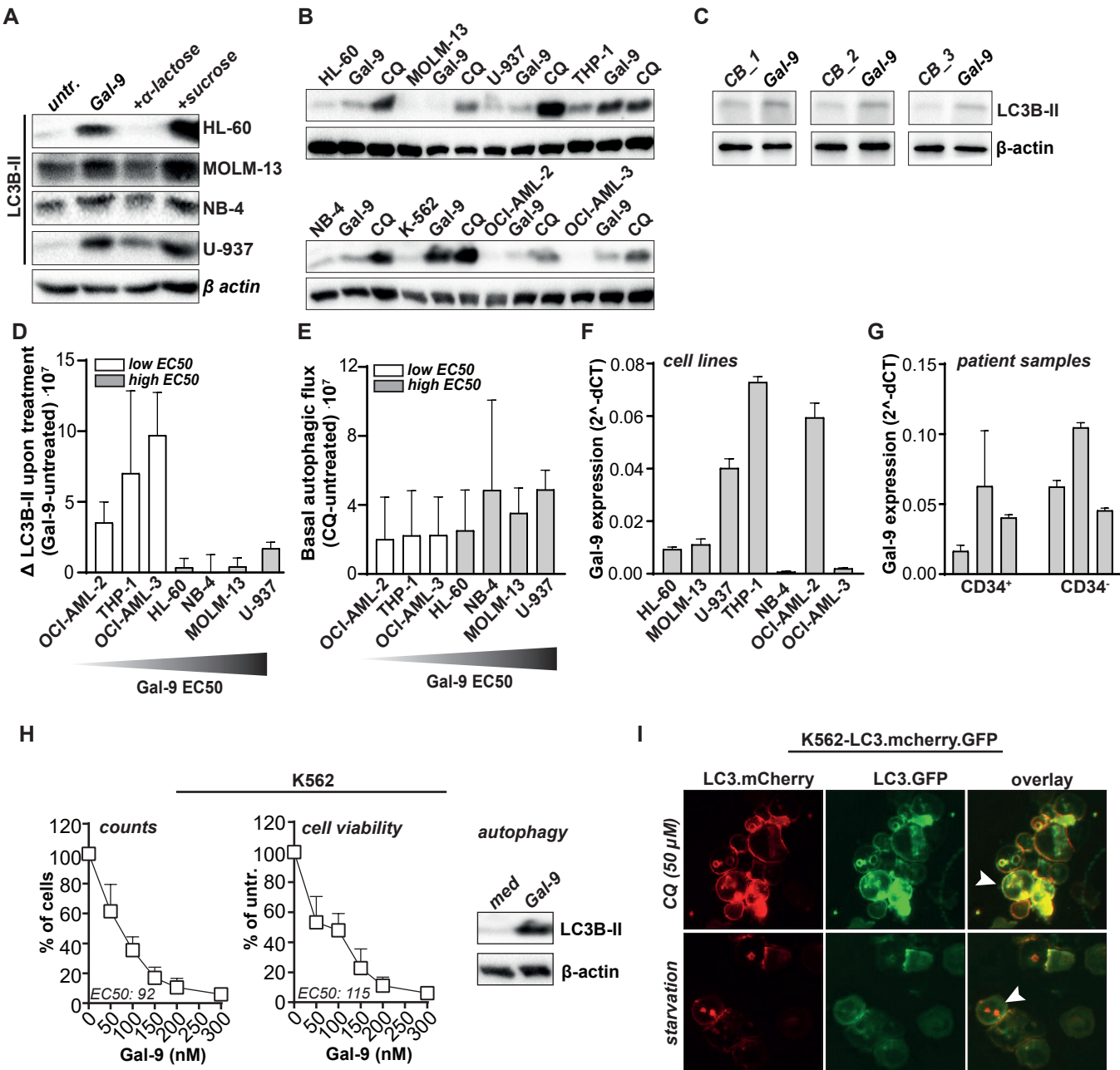

Supplement: Supplementary file 5 — Suppl. Figure 4 [file 41420_2023_1515_MOESM5_ESM.pdf]

Suppl Figure 5

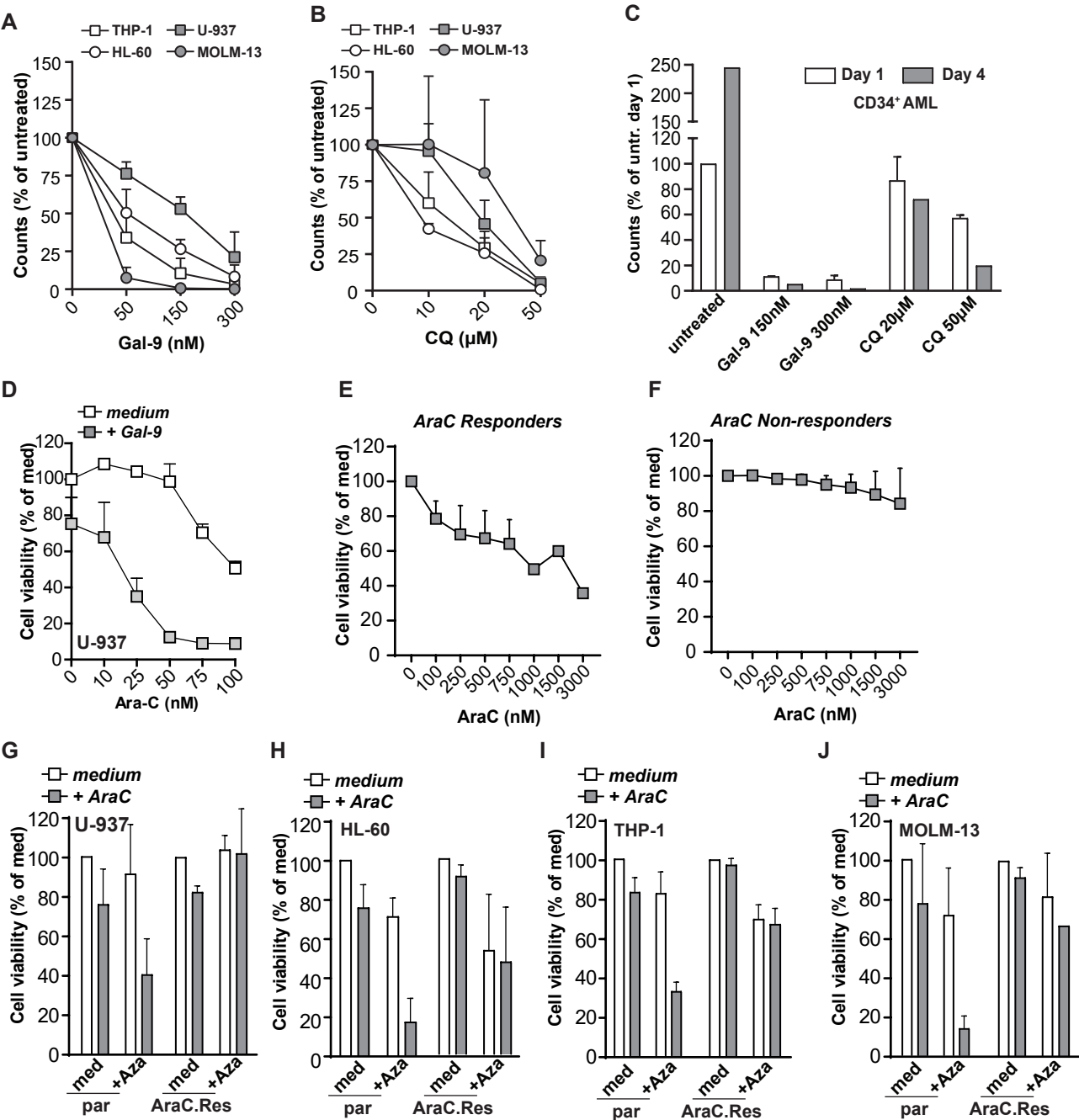

Supplement: Supplementary file 6 — Suppl. Figure 5 [file 41420_2023_1515_MOESM6_ESM.pdf]
