# Supplementary material for: Galectin-9 has non-apoptotic cytotoxic activity toward acute myeloid leukemia independent of cytarabine resistance
Source: Cell Death Discov. 2023 Jul 6;9:228. doi: 10.1038/s41420-023-01515-w (PMC10322858; doi:10.1038/s41420-023-01515-w)
Supplement: Supplementary file 7 — Suppl. Table 1 [file 41420_2023_1515_MOESM7_ESM.pdf]

Suppl Table 1

AML	Age	karyotype	WBC	Risk (ELN17)	FLT3-ITD	EVI1 overexpressie	NPM1	NGS	*in vitro*
1	65	45,XY,-7	54,2	Adverse	none	none	none	EZH2 (0.89), RUNX1 (0.63), IDH2 (0.46)	CD34+ treated with Gal-9
2	65	46,XX,t(11;19)(q23;p13.1)	52,7	Adverse	none	mut	none	ND	
3	68	47,XY,+11	47	Intermediate	none	none	none	ND	
4	60	46,XY,t(7;11)	125,2	Adverse	none	none	none	ND	
5	33	NK	48,9	Adverse	ITD	none	none	ND	
6	78	NK	135,4	Adverse	ITD	none	none	NRAS (0.13), FLT3 (0.23), IDH2 (0.48), SRSF2 (0.63)	CD34- treated with Gal-9
7	34	45,X,-Y	234,7	Intermediate	ITD	none	mut	ND	
8	69	NK	183,8	Favorable	none	none	mut	NPM1 (0.36), FLT3 (0.36), IDH2 (0.40), STAG2 (0.95)	
9	31	46,XY,t(9;11)(p22;q23)	14,4	Adverse	none	mut	none	ND	
10	75	NK	6,6	Favorable	none	none	mut	ND	
11	46	46,Y,t(X;12)(p11.2;q13),t(11;19)(q23;p13.3)	55,4	Adverse	none	none	none	ND	
12	43	NK	23	Favorable	none	none	mut	ND	
13	73	NK	66,7	intermediate	none	none	none	ND	AraC Non-responders
14	73	NK	89,3	intermediate	ITD	none	mut	ND	
15	68	46,XY,t(11;22)(q23;q13)	100,6	Adverse	none	none	none	SRSF2 (0.39), TET2 (0.16)	
16	48	NK	11,7	ND	ND	ND	ND	ND	
17	48	NK	11,7	ND	ND	ND	ND	ND	AraC Responders
18	69	NK	10,8	intermediate	none	none	none	SRSF2 (0.56), ASXL1 (0.06)	
19	61	NK	10,7	intermediate	ITD	none	mut	DNMT3A (0.47), NPM1 (0.38), FLT3 (0.32)	
20	41	NK	23	Adverse	ITD	none	none	ND	
21	71	NK	6,5	Favourable	none	none	none	ND	***Denovo***
22	71							ND	***Relapse***
