# Supplementary material for: Galectin-9 has non-apoptotic cytotoxic activity toward acute myeloid leukemia independent of cytarabine resistance
Source: Cell Death Discov. 2023 Jul 6;9:228. doi: 10.1038/s41420-023-01515-w (PMC10322858; doi:10.1038/s41420-023-01515-w)

Western blots manuscript Choukrani et al

Figure in paper	Sample/ protein	blot name	band #
Figure 3F	caspase 35 kDA	Blot1_Fig3F_casp3-35kDa	all
Figure 3F	caspase 17 kDa	Blot2_Fig3F_casp3-17kDa	all
Figure 4H	THP1_LC3BII	Blot3_Fig4H_Suppl4A_LC3	7-10
Figure 4H	THP1_p62	Blot4_Fig4H_THP1-p62	all
Figure 4H	THP1_actin	Blot5_Fig4H-THP1-actin	7-10
Figure 4H	patient_LC3BII	Blot6_Fig4H-pat-LC3	1-4
Figure 4H	patient_p62	Blot7_Fig4H-pat-p62	1-4
Figure 4H	patient_actin	Blot8_Fig4H-pat-actin	1-4
Figure 4I	THP1-NB4_LC3BII	Blot9_Fig4I-THP1-NB4-LC3BII	all
Figure 4I	THP1-NB4_actin	Blot10_Fig4I-THP1-NB4-actin	all
Figure 4I	MOLM13_OCIAML3_LC3BII	Blot11_Fig4I-MOLM13-OCIAML3-LC3BII	all
Figure 4I	MOLM13_OCIAML3_actin	Blot12_Fig4I-Molm13-OciAml3-actin	1-6
Figure 4I	Pat1_LC3BII	Blot13-Fig4I-patient 1-LC3	last 2
Figure 4I	pat1_actin	Blot14-Fig4I-patient1-actin	last 2
Figure 4I	Pat2_LC3BII	Blot15_Fig4I_Pat2-Pat3_LC3B	last 2
Figure 4I	pat2_actin	Blot16_Fig4I_Pat2-Pat3_actin	last 2
Figure 4I	Pat3_LC3BII	Blot15_Fig4I_Pat2-Pat3_LC3B	first 2
Figure 4I	pat3_actin	Blot16_Fig4I_Pat2-Pat3_actin	first 2
Figure 4I	Pat4_LC3BII	Blot17_Fig4I_Pat4-LC3BII	5,6
Suppl4A	HL60_LC3BII	Blot3_Fig4H_Suppl4A_LC3	1-4
Suppl4A	MOLM13_LC3BII	Blot18_Figsuppl4A-MOLM13-LC3	1-4
Suppl4A	NB4_LC3BII	Blot19_Figsuppl4A-NB4-LC3	2-5
Suppl4A	U937_LC3BII	Blot20_Figsuppl4A-u937-LC3	1-4
Suppl4A	Actin	Blot21_FigSuppl4A-Actin	6-9
Suppl4B	HL60-THP1_LC3BII	Blot22_Figsuppl4B-HL60-THP1-LC3B	1-12
Suppl4B	HL60-THP1_actin	Blot23_Figsuppl4B-HL60-THP1-actin	1-12
Suppl4B	NB4-OCIAML3_LC3BII	Blot24_Figsuppl4B-NB4-OCIAML3-LC3B	1-12
Suppl4B	NB4-OCIAML3_actin	Blot25_Figsuppl4B-NB4-OCIAML3-actin	1-12
Suppl4C	CB_LC3BII	Blot 26_Figsuppl4C-CB-LC3BII	1-2, 4-5, 7-8
Suppl4C	CB_Actin	Blot 27_Figsuppl4C-CB-actin	1-2, 4-5, 7-8
Suppl4H	K562_LC3BII	Blot28_Figsuppl4H-K562_LC3BII	4, 5
Suppl4H	K562_actin	Blot29_Figsuppl4H-K562_actin	4, 5

Blot1_Fig3F_casp3-35kDa

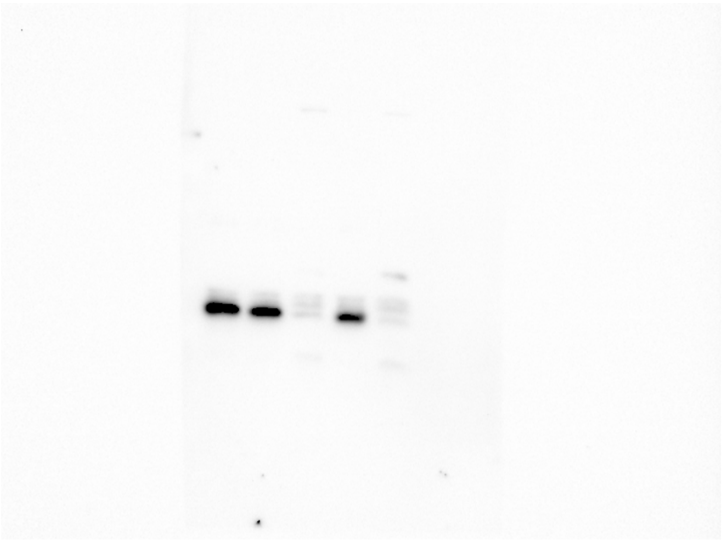

Blot 2_Fig3F_casp3-17kDa

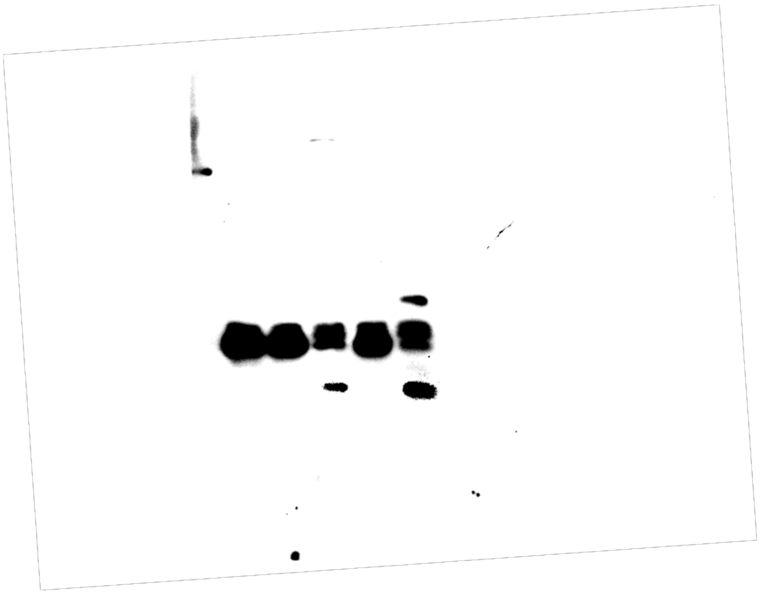

Blot3_Fig4H_suppl4A_LC3

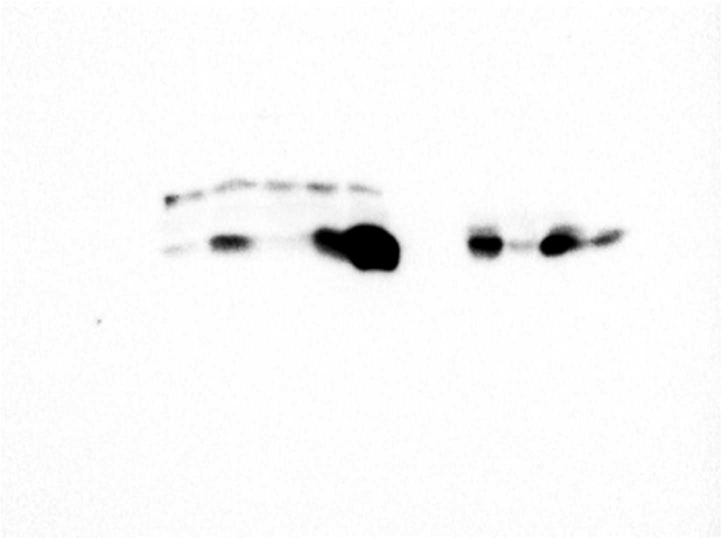

Blot4_Fig4H_THP1-p62

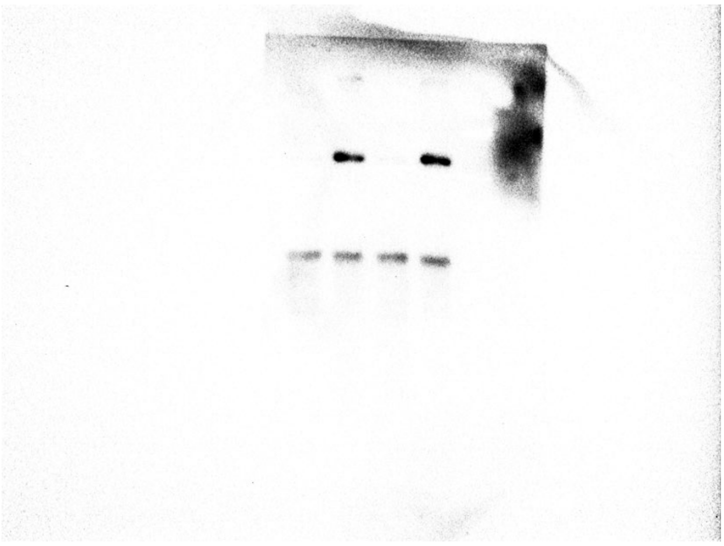

Blot5_Fig4H-THP1-actin

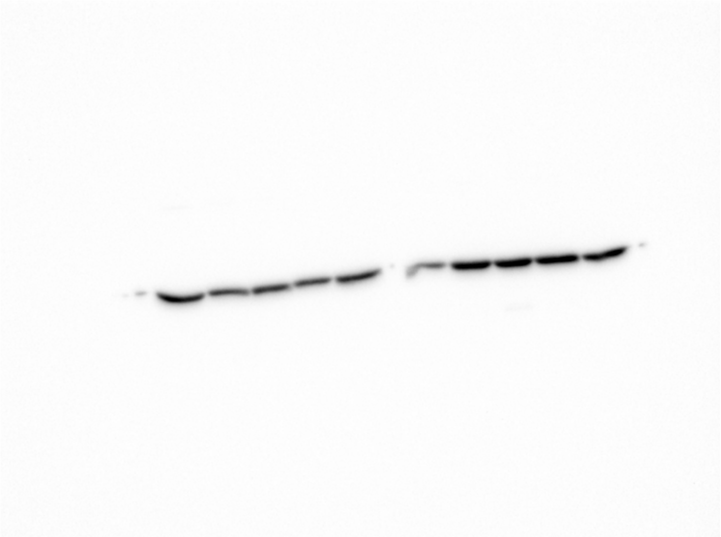

Blot6_Fig4H-pat-LC3

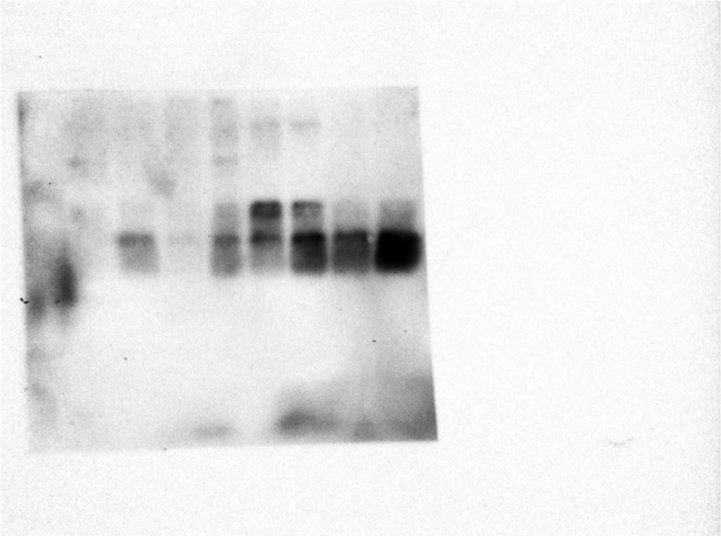

Blot7-Fig4H-pat-62

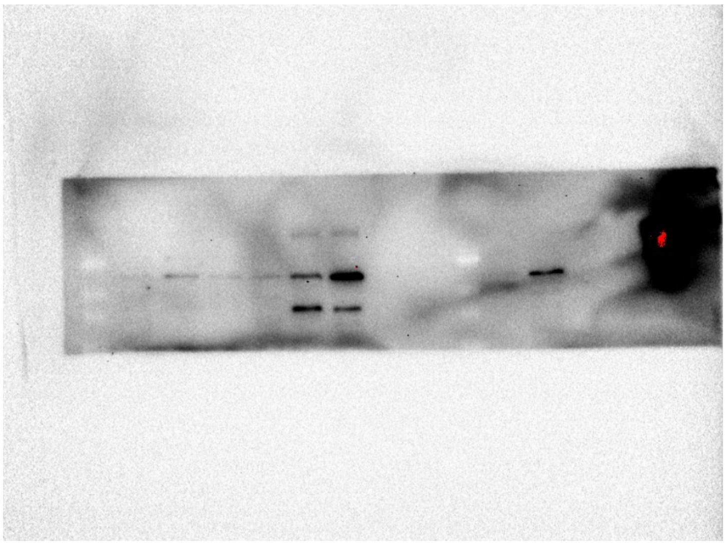

Blot8_Fig4H-pat-actin

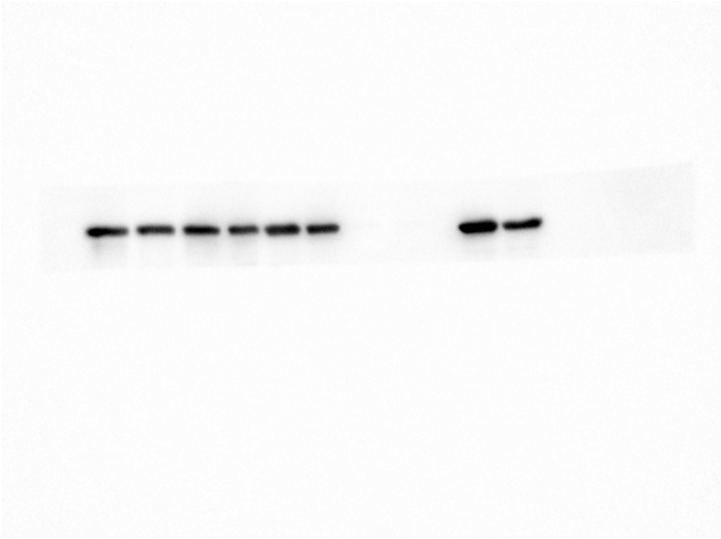

Blot9_Fig4I-THP1-NB4-LC3BII

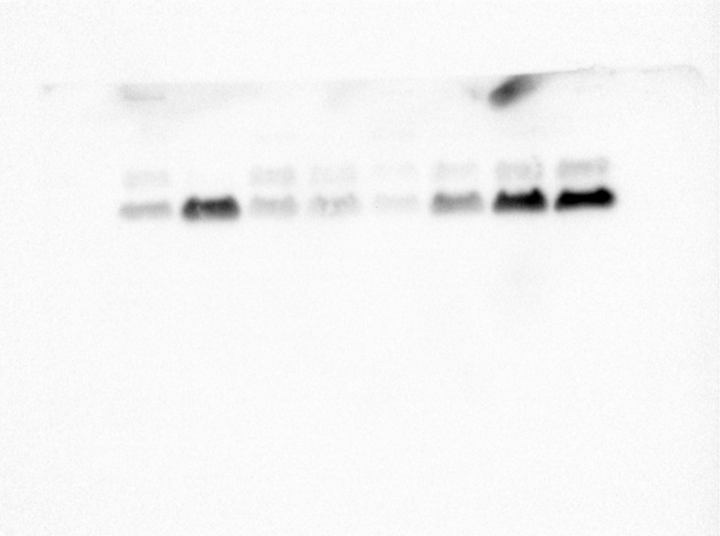

Blot10_Fig4I-THP1-NB4-actin

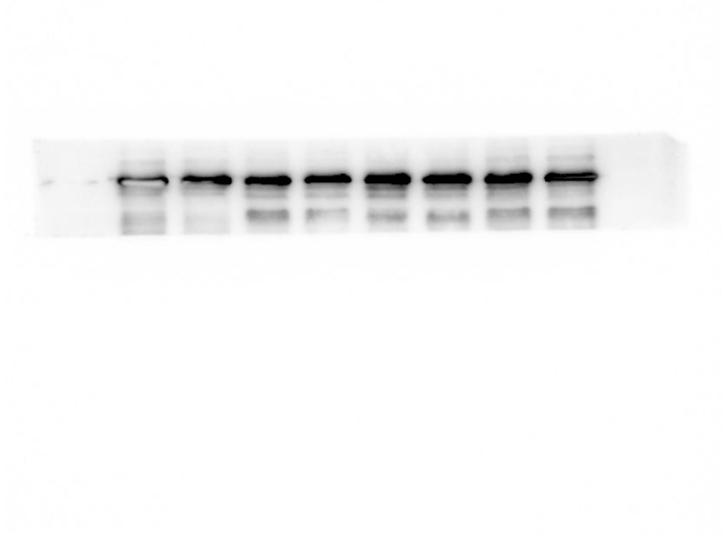

Blot11_Fig4I-MOLM13-OCIAML3-LC3BII

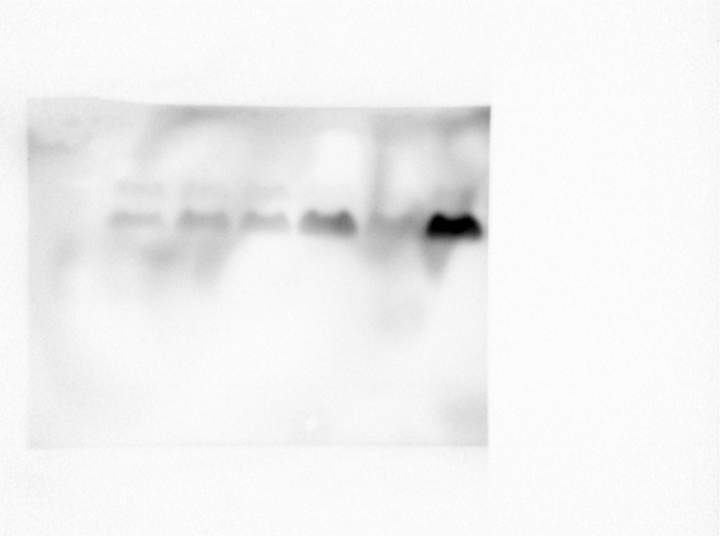

Blot12_Fig4I-Molm13-OciAML3-actin

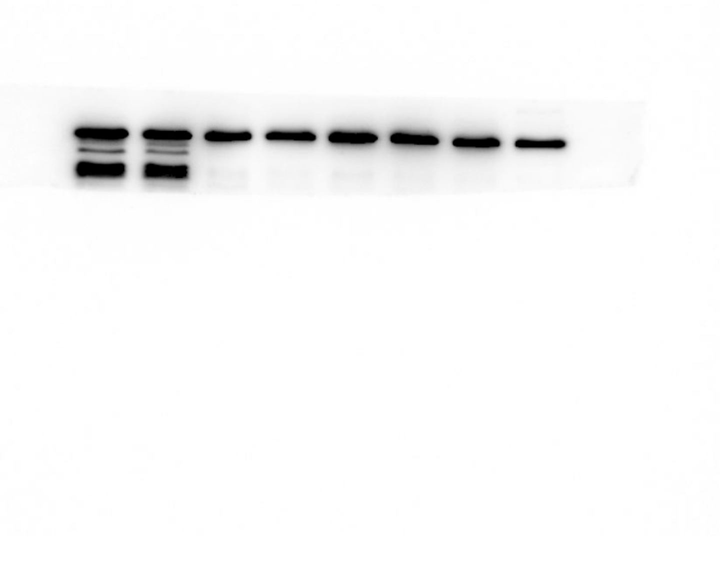

Blot13-Fig4I-patient1-LC3

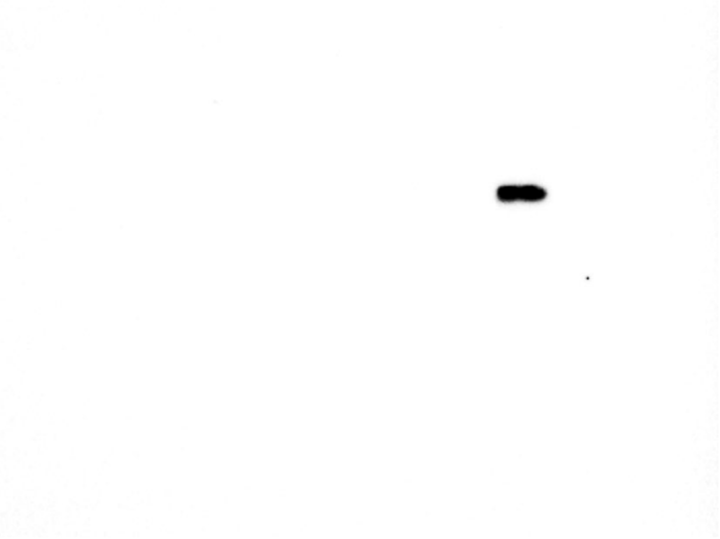

Blot14-Fig4I-patient1-actin

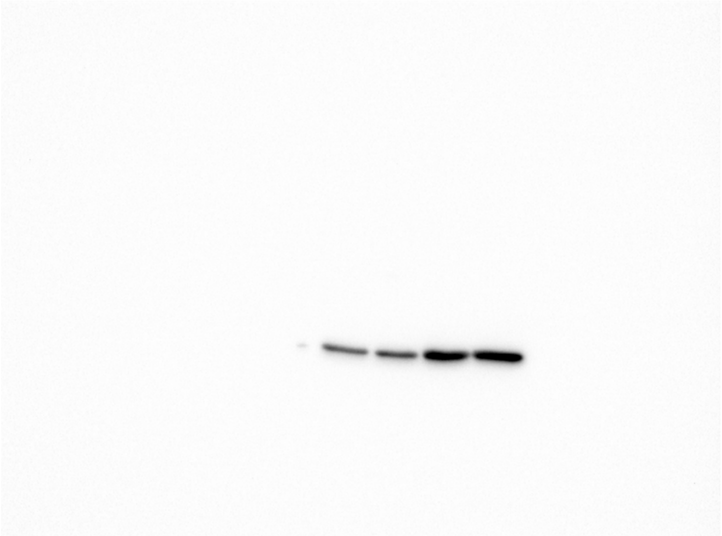

Blot15_Fig4I-Pat2-Pat3_LC3B

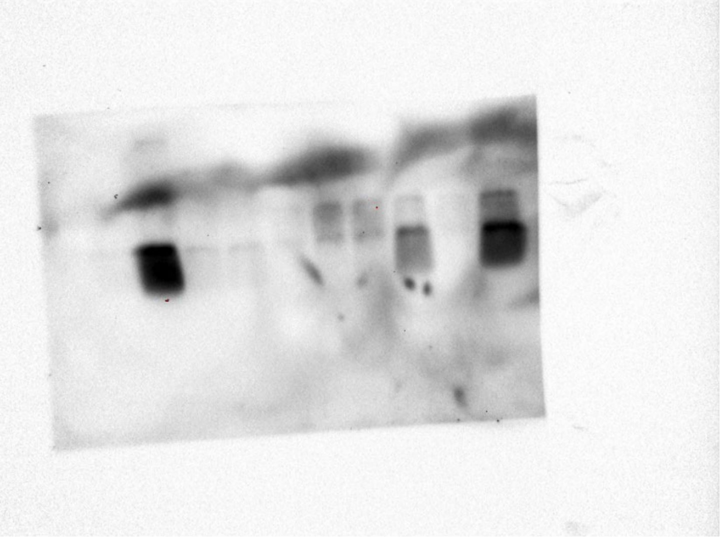

Blot16_Fig4I_Pat2-Pat3_actin

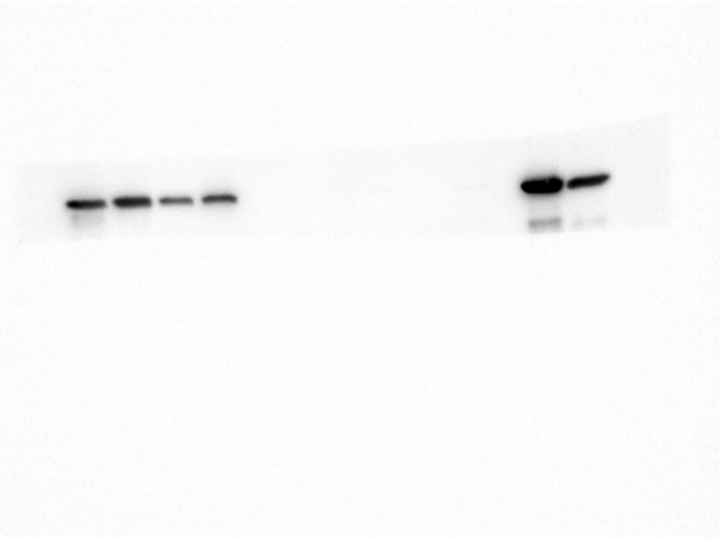

Blot17_Fig4I_Pat4-LC3BII

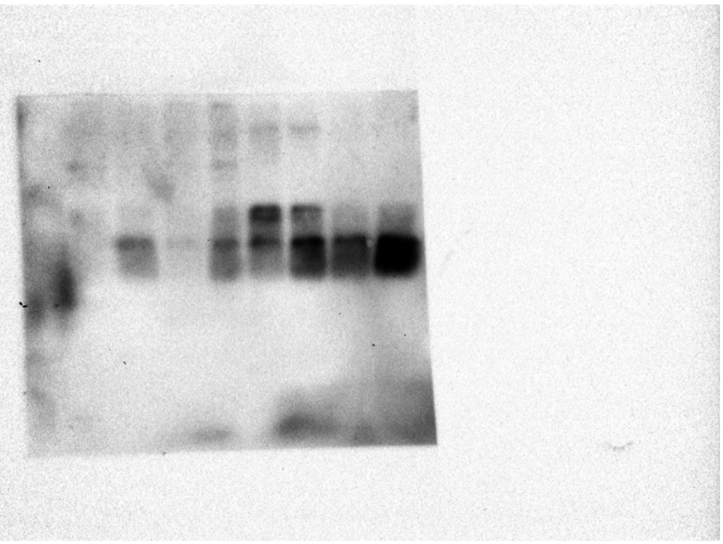

Blot18_Figsuppl4A-MOLM13-LC3

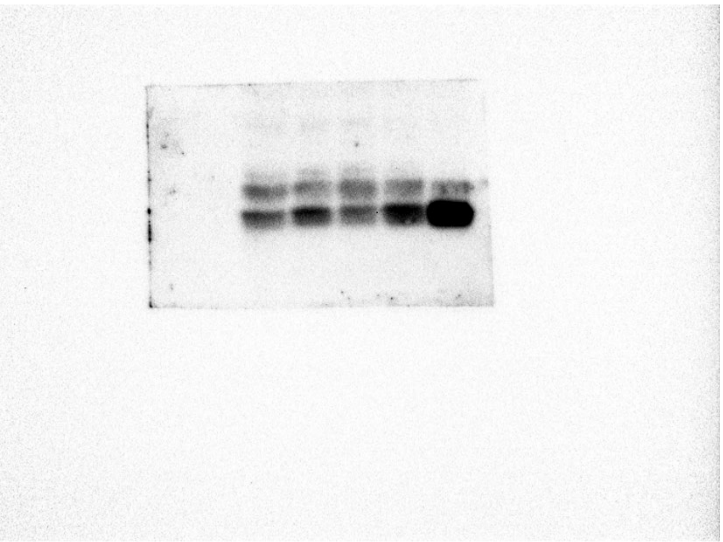

Blot19-Figsuppl4A-NB4-LC3

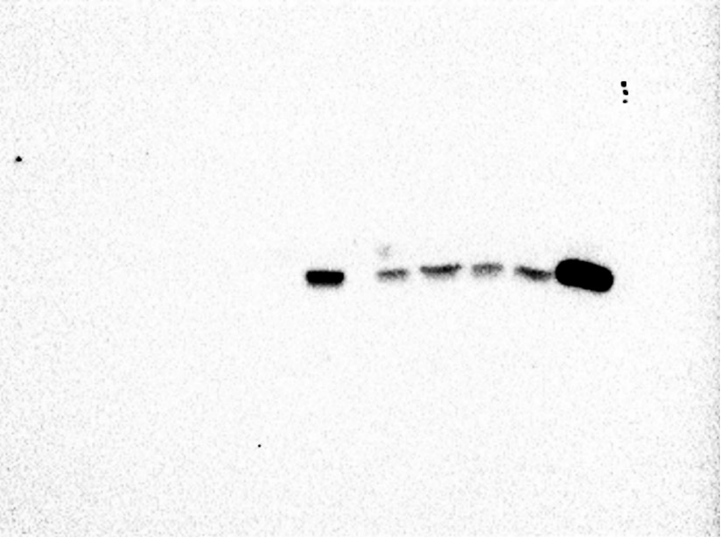

Blot20_Figsuppl4A-U937-LC3

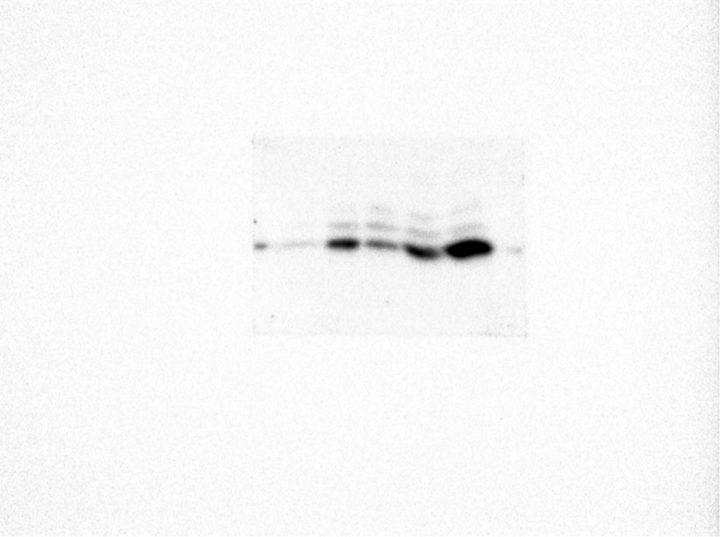

Blot21_FigSuppl4A-Actin

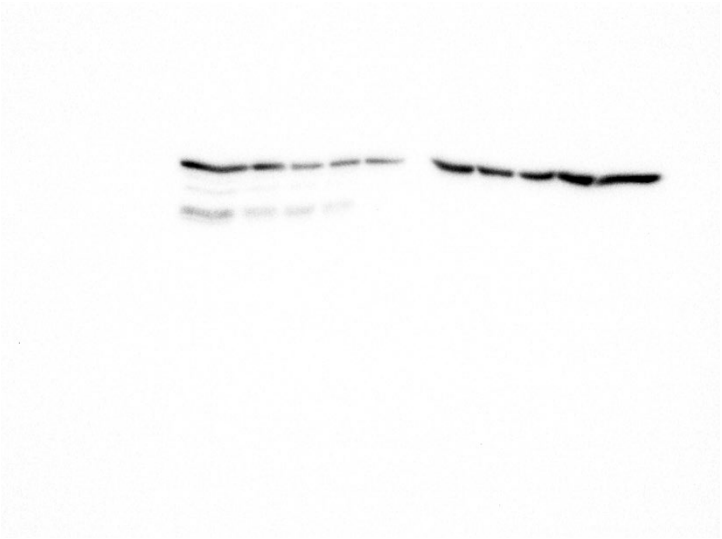

Blot22_Figsuppl4B-HL60-THP1-LC3B

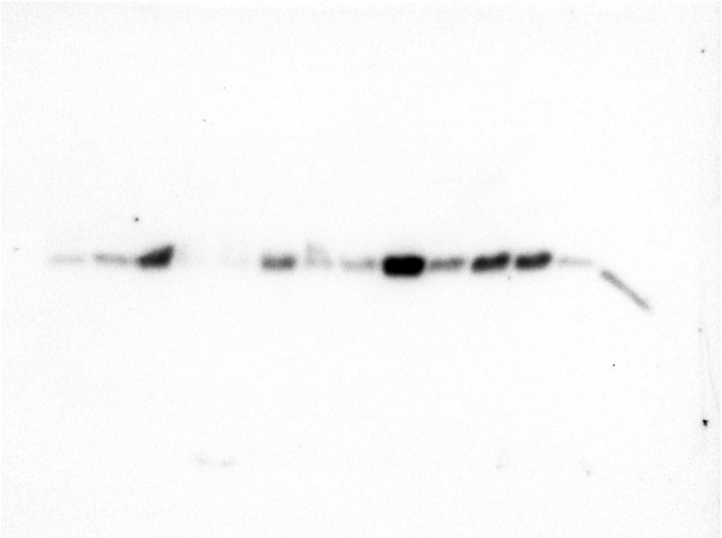

Blot23_Figsuppl4B-HL60-THP1-actin

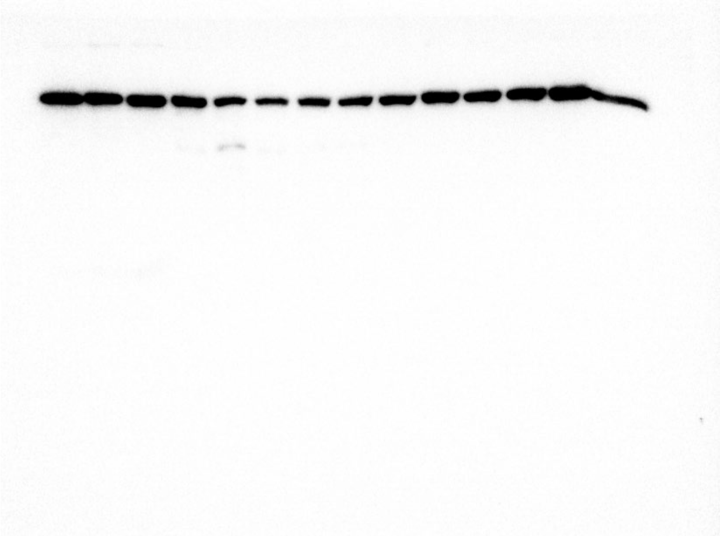

Blot24_Figsuppl4B-NB4-OCIAML3-LC3B

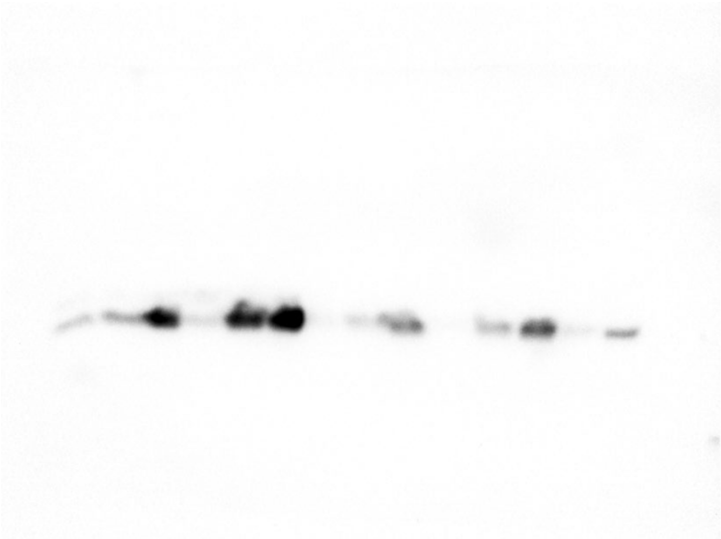

Blot25_Figsuppl4B-NB4-OCIAML3-actin

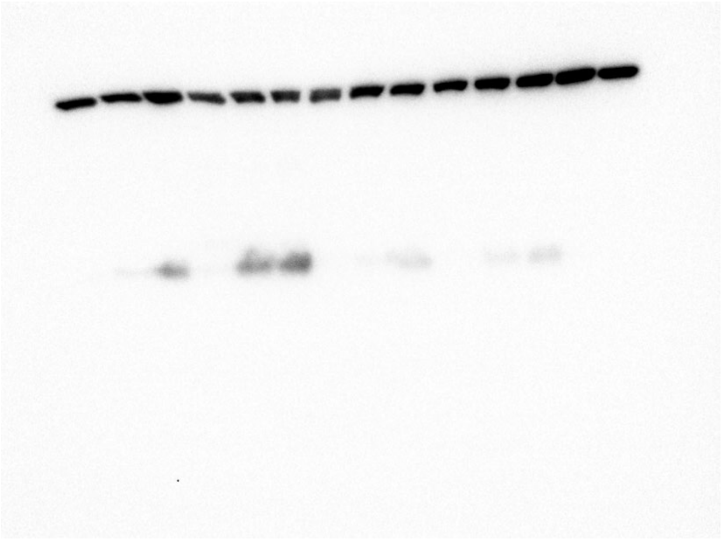

Blot 26_Figsuppl4C-CB-LC3BII

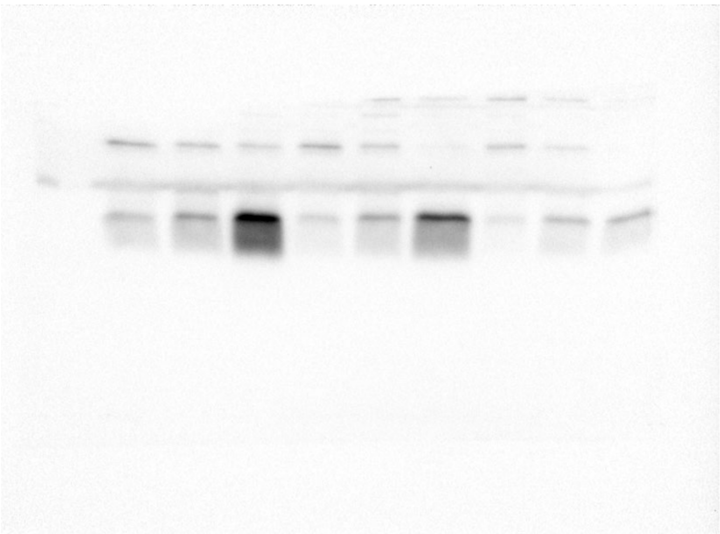

Blot 27_Figsuppl4C-CB-actin

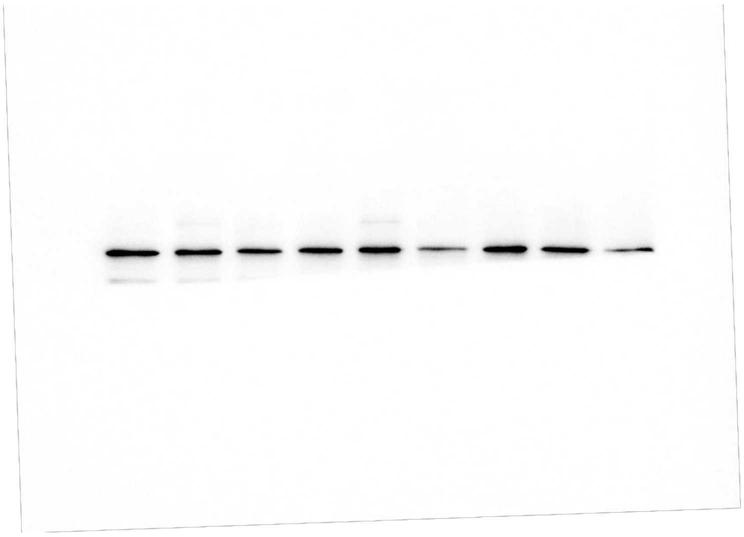

Blot28_Figsuppl4H-K562_LC3BII

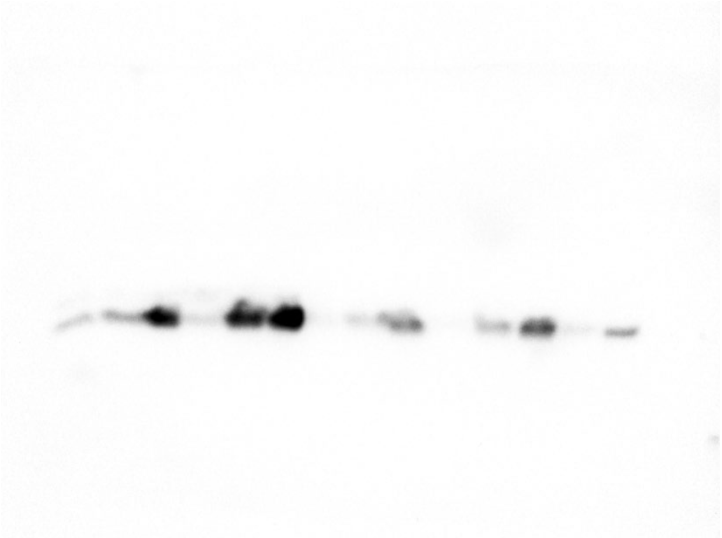

Blot29_Figsuppl4H-K562_actin

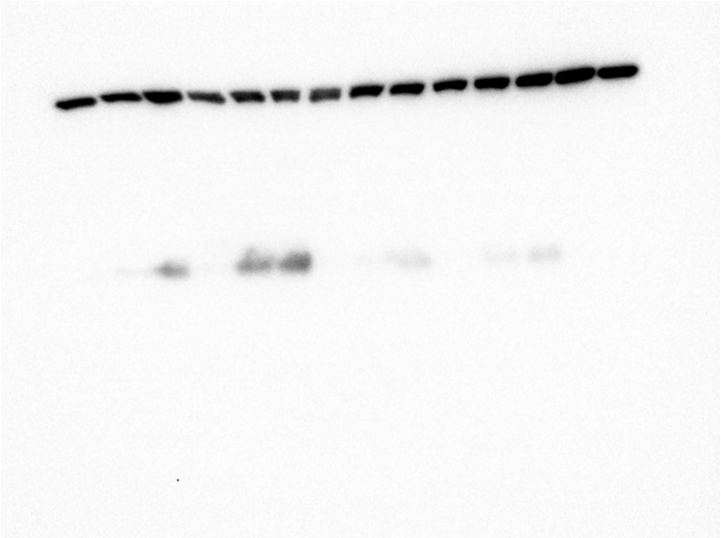

Supplement: Supplementary file 8 — Uncropped western blots [file 41420_2023_1515_MOESM8_ESM.pdf]
